# Structural basis and mode of action for two broadly neutralizing nanobodies targeting the highly conserved spike stem-helix of sarbecoviruses including SARS-CoV-2 and its variants

**DOI:** 10.1371/journal.ppat.1013034

**Published:** 2025-04-11

**Authors:** Liyan Guo, Zimin Chen, Sheng Lin, Fanli Yang, Jing Yang, Lingling Wang, Xindan Zhang, Xin Yuan, Bin He, Yu Cao, Jian Li, Qi Zhao, Guangwen Lu

**Affiliations:** 1 Department of Emergency Medicine, State Key Laboratory of Biotherapy, West China Hospital, Sichuan University, Chengdu, Sichuan, China; 2 Department of Hematology, West China Hospital, Sichuan University, Chengdu, Sichuan, China; 3 Disaster Medicine Center, West China Hospital, Sichuan University, Chengdu, Sichuan, China; 4 School of Basic Medical Sciences, Chengdu University, Chengdu, Sichuan China; 5 College of Food and Biological Engineering, Chengdu University, Chengdu, Sichuan China; Loyola University Chicago Stritch School of Medicine, UNITED STATES OF AMERICA

## Abstract

The persistent emergence of new severe acute respiratory syndrome coronavirus 2 (SARS-CoV-2) variants highlights the need for developing broad-spectrum antiviral agents. Here, we report the identification of two sarbecovirus S2-specific alpaca nanobodies, namely H17 and H145, that effectively neutralize known SARS-CoV-2 variants (including the Omicron subvariants) and other sarbecoviruses (such as SARS-CoV, PANG/GD, WIV1, and HKU3). The two nanobodies recognize a linear epitope (D_1139_PLQPELDSFKEEL_1152_) in the upper region of the S2 stem-helix (SH), which is highly conserved among SARS-CoV-2 variants and other sarbecoviruses. The complex structure of the nanobody bound to the epitope SH-peptide reveal that nanobody binding will impede the refolding of S2, effectively neutralizing the virus. Moreover, the nanobodies bind viral S2 in an acidification-insensitive manner, demonstrating their capacity for entry inhibition especially when viruses enter via the endosomal route. Finally, H17 and H145 possess a better taking-action window for virus neutralization, superior to the RBD-targeting nanobodies that exert neutralization by competing against ACE2 binding. Taken together, the results suggest that anti-SH nanobodies H17 and H145 are promising broad-spectrum drug candidates for preventing and treating the pandemic infections by SARS-CoV-2 variants and other sarbecoviruses.

## Author summary

Nanobody, as the smallest functional antibody fragment, is an important group of viral inhibitors. In this study, we identified two neutralizing nanobodies, H17 and H145, that targeted the highly conserved spike (S) stem-helix in SARS-CoV-2. The two nanobodies effectively neutralized known SARS-CoV-2 variants and a range of other sarbecoviruses. Structural analyses revealed that the two nanobodies would likely prevent the postfusion S formation by impeding S2 refolding for neutralization. In addition, the nanobody binding was acidification-insensitive, offering strong entry inhibition particularly when the virus entered via the endosomal route. Finally, we showed that H17 and H145 possessed a better taking-action window for virus neutralization when compared to RBD-targeting nanobodies. These data suggest that the two nanobodies could serve as an effective drug reserve against sarbecoviruses including SARS-CoV-2.

## Introduction

The coronavirus disease 2019 (COVID-19) pandemic, caused by severe acute respiratory syndrome coronavirus 2 (SARS-CoV-2), has profoundly impacted public health and the global economy. Coronaviral entry into host cells, facilitated by the spike (S) glycoprotein, is the initial step in infection. This process involves the S1 subunit binding to the host cell receptor and the S2 subunit driving membrane fusion [1]. The receptor-binding domain (RBD) within the S1 subunit interacts with the angiotensin-converting enzyme 2 (ACE2) receptor on host cells [2]. The S1-RBD is a critical antigenic component for the development of neutralizing antibodies (nAbs) and vaccines. Thus, considerable efforts have incorporated the S1-RBD as a component or as the sole antigen to create high-efficacy nAbs, vaccines, and antiviral agents [3–5]. However, RBD is poorly conserved. The rapid evolution of SARS-CoV-2 has clustered multiple mutations within S, mainly in RBD. For example, the Omicron S protein contains more than 30 mutations, most of which are located in S1-RBD [6]. These mutations are found in a series of circulating strains, including BA.1, BA.2, BA.4/5, BA.2.12.1, XBB.1.5, XBB.1.16, EG.5.1, BA.2.86, JN.1, and KP.2 [7–9]. Notably, these mutations have resulted in an altered antigenic structure, leading to severe immune evasion and thus dramatically compromising the efficacy of RBD-targeting nAbs [10–13]. This has posed continuous challenges for the prevention and treatment of COVID-19. In addition to SARS-CoV-2, the *Sarbecovirus* subgenus also contains other zoonotic coronaviruses isolated from bats, pangolins, and palm civets [14]. These zoonotic sarbecoviruses are able to recognize the host receptor ACE2 or its orthologues, raising concerns for a potential pandemic caused by these coronaviruses via cross-species transmission [15,16]. Though the viral S of these sarbecoviruses exhibits overall a high degree of sequence homology, the S1 subunit, especially the S1-RBD region, is the least conserved. Accordingly, the majority of existing SARS-CoV-2 neutralizing antibodies exhibit narrow neutralization breadth against these zoonotic coronaviruses. It is therefore imperative to explore broad-spectrum pan-sarbecovirus nAbs that can recognize conserved cross-reactive epitopes.

In comparison to S1-RBD, the fusion-driven S2 is much more conserved in both sequence and structure. During S2-mediated membrane fusion, the heptad repeat 1 (HR1) undergoes a jack-knife transition to insert the fusion peptide into the target membrane. It links with the central helix (CH) to form an elongated HR1-CH three-helices. Simultaneously, three outer-helices composed of the heptad repeat 2 (HR2) and the stem-helix (SH), initially fold back and tightly fit into the groove of the HR1-CH coiled-coil in an antiparallel manner. Resultantly, HR2 binds to HR1 to form the six-helix bundle (6HB) structure, and SH combines with the outer-region of CH to assist 6HB formation, leading to a very rigid fusion-core structure [17,18]. While S1-RBD is relatively divergent in sequence, the sequence identity of HR1, HR2, and SH in S2 is more than 90% across different sarbecoviruses [19]. Peptides and antibodies that specifically target the S2 fusion core show superior broad-spectrum antiviral activity [20–25]. These findings suggest that the S2 fusion core is a very promising target for developing cross-variant and even pan-sarbecovirus antibodies.

Nanobodies are natural, single antigen-binding domains of heavy chain-only antibodies. They are the smallest functional antibody fragments (<15 kDa) that can specifically recognize antigen epitopes with high affinity; thus, they are more likely to reach the cryptic epitopes that are usually inaccessible to conventional human antibodies [26]. Furthermore, nanobodies have unique characteristics compared to traditional monoclonal antibodies (mAbs), such as superior stability, greater water solubility, easy for engineering, lower immunogenicity, higher yields, and cost-effective manufacturing [26]. Therefore, nanobodies have become a desirable candidate for next-generation intervention and treatment of respiratory viral infections. Previous studies have reported the development of multiple neutralizing nanobodies (nNbs) against SARS-CoV-2 [27–30]. Nevertheless, the majority of these nNbs specifically target the S1-RBD region and are easily escaped.

In this study, we isolated two broad neutralizing nanobodies (namely H17 and H145) from an alpaca that had been immunized with the trimeric SARS-CoV-2 S2 fusion-core-based antigen. Both nanobodies could neutralize the infection by a wide range of sarbecoviruses, including the predominant SARS-CoV-2 variants (especially the Omicron subvariants), Severe acute respiratory syndrome (SARS-CoV), PANG/GD, WIV1, and HKU3. Notably, they recognized a liner epitope spanning D_1139_PLQPELDSFKEEL_1152_ in the upper region of SH. The epitope was highly conserved among SARS-CoV-2 variants and other sarbecoviruses, indicating that the neutralizing capacity of the two nanobodies would not be susceptible to the known mutations in SARS-CoV-2 variants. Structurally, H17 and H145 bound to the unexposed inner face of SH rather than to the exposed helical region. When binding to viral S, they would disrupt the prefusion S-conformation and/or block the formation of S2 fusion machinery in the post-fusion S-conformation, thus preventing viral-host membrane fusion. Moreover, our results showed that H17 and H145 could bind to viral S in an acidification-insensitive manner, implying that their entry-inhibitory activity would remain unaffected when the virus entered via the endosomal pathway. Furthermore, H17 and H145 possessed a longer taking-action window than the RBD-targeting nNbs, suggesting that they might be developed, as drug candidates, for prevention and treatment of the ongoing COVID-19 pandemic dominated by SARS-CoV-2 variants and the potential cross-species infections by other sarbecoviruses.

## Results

### Identification of neutralizing nanobodies targeting SARS-CoV-2 S2

**Fig 1:**
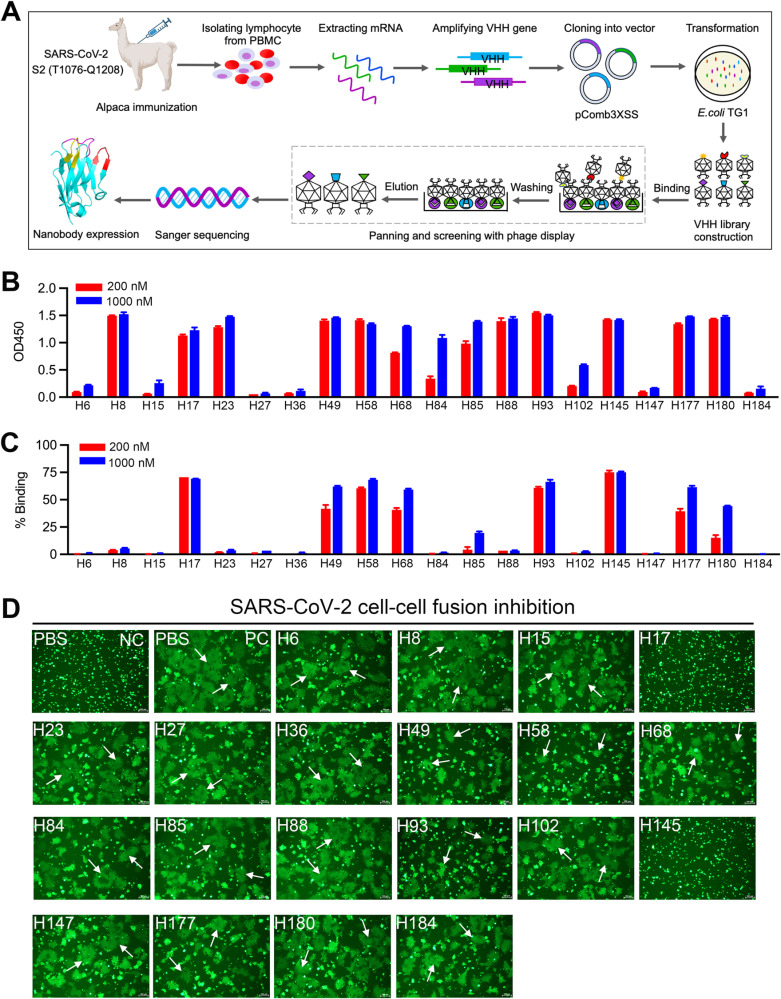
Screening and identification of SARS-CoV-2 S2-specific neutralizing nanobodies. (A) Schematic diagram showing the construction of the VHH phage library specific for the SARS-CoV-2 S2 (T1076-Q1208) antigen. The process includes immunizing an alpaca with the SARS-CoV-2 S2 (T1076-Q1208) antigen, isolating lymphocytes from peripheral blood mononuclear cells (PBMC), constructing a VHH phage library, panning and screening S2-specific VHH phages, Sanger sequencing, and VHH protein expression. This figure was created using BioRender.com. (B) ELISA-binding profiles of the S2 (T1076-Q1208) protein and the indicated nanobodies. Emission OD450 values are plotted as histograms. (C) Binding capacity of the indicated nanobodies to the SARS-CoV-2 S protein on the surface of HEK-293T cells in flow cytometry. (D) Inhibition of SARS-CoV-2 S-mediated syncytium formation in the presence of the indicated nanobodies. NC, negative control: only HEK-293T/EGFP/S cells. PC, positive control: syncytia formation induced by mixing HEK-293T/EGFP/S cells with HEK-293T-hACE2 cells in the absence of nanobodies. Fused syncytia are indicated with white arrows. Scale bar is 100 μm.

To identify nanobodies targeting the highly conserved region in the SARS-CoV-2 S2 subunit, we initially designed a trimeric S2 fusion-core-based antigen, namely S2 (T1076-Q1208), that involved both the SH and HR2 components. The antigen was successfully prepared with high purity (S1 Fig). We subsequently vaccinated an alpaca with the S2 (T1076-Q1208) antigen and then constructed a variable domain of heavy chain (VHH) phage library (**Fig 1A**). The antigen-specific candidate clones were identified and sequenced (S2 Fig). A total of 20 high-purity nanobodies were finally prepared via the *Brevibacillus* expression system (S3 Fig).

The binding of these prepared nanobodies to the S2 (T1076-Q1208) protein was assessed using an enzyme-linked immunosorbent assay (ELISA), and their binding to the full-length viral S protein was evaluated by flow cytometry. A total of 13 nanobodies (H8, H17, H23, H49, H58, H68, H84, H85, H88, H93, H145, H177, and H180) showed high binding potency to S2 (T1076-Q1208) in ELISA (**Fig 1B**). Additionally, 8 nanobodies (H17, H49, H58, H68, H93, H145, H177, and H180) exhibited S-binding capacity in flow cytometry. Among these, 4 nanobodies (H17, H58, H93, and H145) showed strong S-binding capabilities (**Fig 1C**).

To identify the most effective anti-S2 neutralizing nanobodies, their ability to inhibit syncytium formation was assessed using a SARS-CoV-2 S-mediated cell-cell fusion assay. The results showed that two nanobodies, H17 and H145, exhibited optimal fusion-inhibition activity (**Fig 1D**). These findings suggest that H17 and H145 are efficacious neutralizing nanobodies by targeting SARS-CoV-2 S2.

### A linear epitope in the upper region of SARS-CoV-2 S2 stem-helix recognized by H17 and H145

To identify the epitopes recognized by H17 and H145, we assessed their binding ability to a series of truncated S2 proteins using Western Blot (WB), ELISA, and Bio-Layer Interferometry (BLI). This allowed us to pinpoint the shortest epitope recognized by the two nanobodies. Initially, a range of truncated proteins, fused with glutathione S-transferases (GST) at the N-terminus, were designed and prepared (S4 Fig). We first evaluated the binding of H17 and H145 to the denatured antigen-truncates by WB. Both nanobodies reacted strongly with the denatured antigens, suggesting that they recognized a linear epitope composed of a consecutive sequence of amino acids. The shortest antigen-truncate that could react with H17 and H145 was S2 (D1139-L1152) (Figs S5A, 2A and 2B). Next, we measured the binding of H17 and H145 to the non-denatured antigen proteins via ELISA. The results showed that both nanobodies could bind to the shortest antigen, S2 (D1139-L1152), with EC50s of 11.31 nM for H17 and 2.00 nM for H145, respectively (Figs S5B, S5D, 2C, 2D and 2G). Echoing our WB and ELISA results, the BLI assay also showed that the shortest antigen S2 (D1139-L1152) readily bound to H17 and H145 (Figs S5C, 2E and 2F). Collectively, the WB, ELISA, and BLI results demonstrate that H17 and H145 recognize a linear epitope spanning D_1139_PLQPELDSFKEEL_1152_ in SARS-CoV-2 S2 SH.

**Fig 2:**
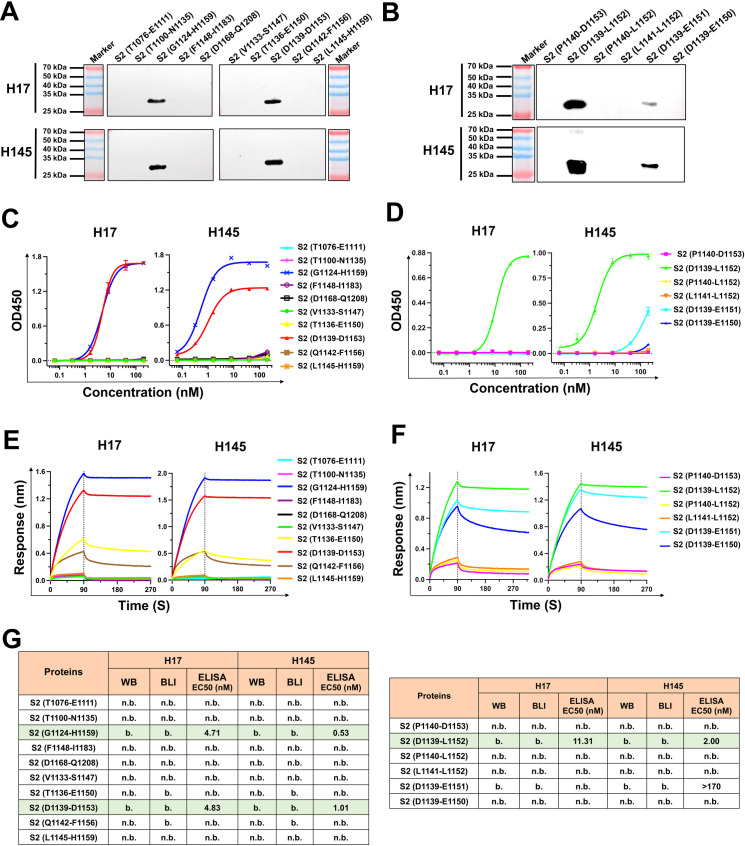
Identification of epitopes recognized by nanobodies H17 and H145. (A-B) The binding ability of H17 and H145 to a series of truncated S2 proteins which fused with GST at the N-terminus using Western Blot (WB). (C-D) Multi-concentration ELISA-binding profile of H17 and H145 to the indicated S2 antigen. OD450 values are plotted as curves. Data are means ± SD of triplicate samples. (E-F) Binding ability of the indicated S2 antigen to H17 and H145 analyzed by biolayer interferometry (BLI). Biotinylated H17 and H145 were immobilized to SA chip. Single association and dissociation curves were detected. b., binding. n.b., no binding. (G) Summary of the binding features of H17 and H145 to the denatured antigen-truncates by WB, ELISA, and BLI.

Notably, both H17 and H145 showed dramatically decreased binding to S2 (D1139-E1151). In ELISA, H17 hardly bound to S2 (D1139-E1151) and H145 bound to S2 (D1139-E1151) with more than 86.5 times lower affinity than to S2 (D1139-L1152) (Fig 2D). In BLI, both S2 (D1139-E1151) and S2 (D1139-E1150) displayed, when compared to S2 (D1139-L1152), slower association-rate and faster dissociation-rate (Fig 2F). These observations indicated that the amino acid E1151 was crucial for the epitope binding by H17 and H145. Additionally, neither H17 nor H145 bound to S2 (P1140-D1153), S2 (P1140-L1152), or S2 (L1141-L1152) (Fig 2D and 2F), suggesting that D1139 was also a key residue for the recognition by the two nanobodies.

### Broad neutralizing capacity of H17 and H145 against SARS-CoV-2 variants

**Fig 3:**
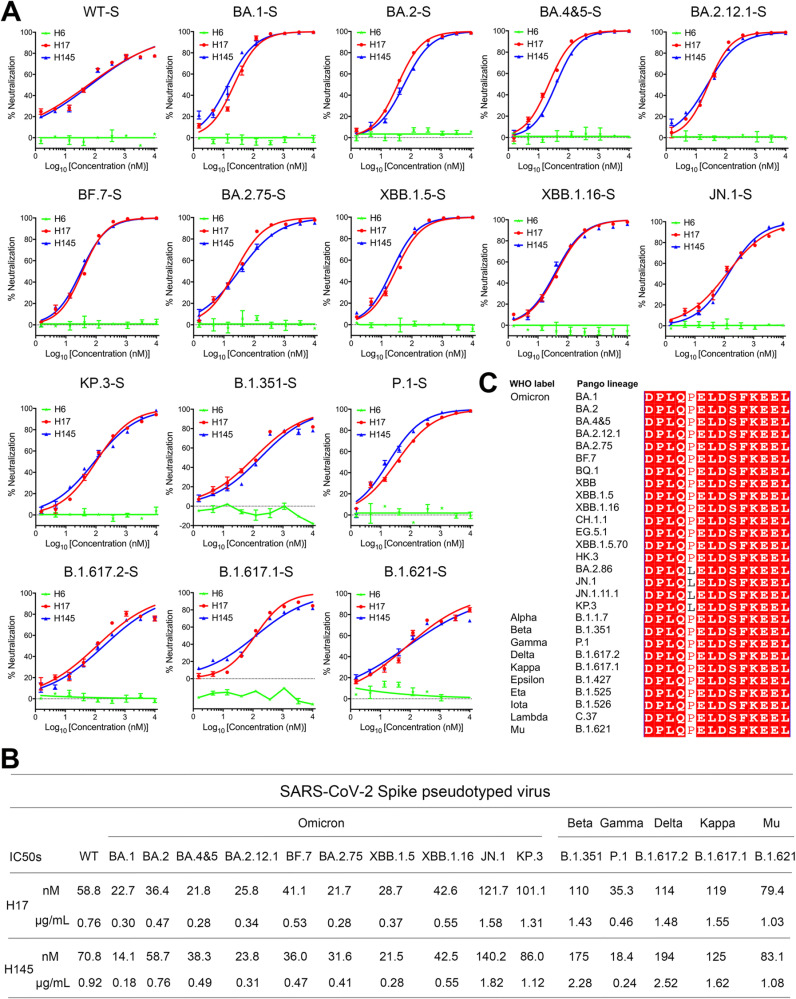
Neutralizing efficacy of H17 and H145 agaisnt SARS-CoV-2 variants. (A) Cross-neutralization activity of H17 and H145 against the SARS-CoV-2 prototype and its major variants in the pseudovirus neutralizing assay. H6 was used as a negative control. Data are expressed as means ± SD. Experiments were performed in triplicate. (B) Table summarizing the neutralizing IC50 values. (C) Sequence conservation of the stem helix epitope spanning DPLQPELDSFKEEL among diverse SARS-CoV-2 variants. The sequence corresponds to spike residues 1139 to 1152 of the SARS-CoV-2 prototype virus. Sequences were downloaded from the GISAID database and https://covariants.org.

As the global spread of SARS-CoV-2 continues, numerous variants have emerged. These include the known VOC (Variants of Concern) strains such as Alpha, Beta, Gamma, Delta, and Omicron, as well as various VOI (Variants of Interest) strains such as Kappa and Mu. These variants have led to repeated outbreaks of the SARS-CoV-2 pandemic, drawing global attention. The accumulated mutations in the S protein, especially those in the RBD region, contribute significantly to immune evasion. Subsequently, several neutralizing antibody drugs in market completely lost efficacy [10,31]. To evaluate the virus entry-inhibition efficacy of H17 and H145 against SARS-CoV-2 and its variants, we performed pseudovirus neutralization assay. Echoing the results of our syncytium-formation inhibition assay, H17 and H145 effectively inhibited the wild-type(WT)-S-mediated pseudovirus infection, with half-maximal inhibitory concentration (IC50) values of 58.8 nM for H17 and 70.8 nM for H145, respectively (Fig 3A and 3B). Additionally, both nanobodies were able to cross-neutralize the infection by known SARS-CoV-2 variants with high potency. Targeting the Omicron subvariants, including BA.1, BA.2, BA.4&5, BA.2.12.1, BF.7, BA.2.75, XBB.1.5, and XBB.1.16, the IC50 values were determined to range from 21.7 to 42.6 nM for H17 and 14.1 to 58.7 nM for H145, respectively. We also find that the S proteins of the newly circulating variants of JN.1 and KP.3 carry the P1143L mutation in the S2 (D1139-L1152) epitope region. We therefore further performed experiments to characterize the neutralization capacities of our nanobodies against these two variants. The results showed that both nanobodies were able to effectively inhibit the entry of JN.1 and KP.3 pseudoviruses (with IC50s of 121.7 nM and 101.1 nM for H17, and 140.2 nM and 86.0 nM for H145, respectively), though with somewhat reduced neutralizing activity compared to the earlier Omicron variants. For pre-Omicron variants, including Beta (B.1.351), Gamma (P.1), Delta (B.1.617.2), Kappa (B.1.617.1), and Mu (B.1.621), the IC50 values were calculated to range from 35.3 to 119.0 nM for H17 and 18.4 to 194.0 nM for H145, respectively (Fig 3A and 3B).

We further compared the epitope sequences in these SARS-CoV-2 variants. The results showed that this epitope is highly conserved among thus-far reported Omicron subvariants (including BA.1, BA.2, BA.4&5, BA.2.12.1, BA.2.75, BF.7, BQ.1, XBB, XBB.1.5, XBB.1.16, CH.1.1, EG.5.1, XBB.1.5.70, and HK.3), as well as the pre-Omicron variants (including Alpha, Beta, Gamma, Delta, Kappa, Epsilon, Eta, Iota, Lambda, and Mu). Though the recently immune-evading Omicron mutants, including BA.2.86, JN.1, JN.1.11.1, and KP.3, contain the P1143L mutation within the SH epitope, the mutation does not significantly influence the antiviral efficiency of H17 and H145. We also retrieved SARS-CoV-2 spike protein sequences from the NCBI database and removed erroneous sequences using MEGA7 software [32], resulting in a final dataset of 7,107 protein sequences. The SH amino acid sequence of N1135-V1164 were compared using WebLogo [33]. The results showed that, at position 1143, the relative frequency of leucine (L) is approximately 0.7 and proline (P) occurs at a frequency of approximately 0.3. Residues D1139, S1147, K1149, and E1151 also show mutations but with a negligible frequency of ~0.001 (S6 Fig). Overall, conservation of the SH-epitope region recognized by H17 and H145 explains their broad neutralizing activites (**Fig 3C**).

### Cross-neutralization of sarbecovirus infection by H17 and H145

**Fig 4:**
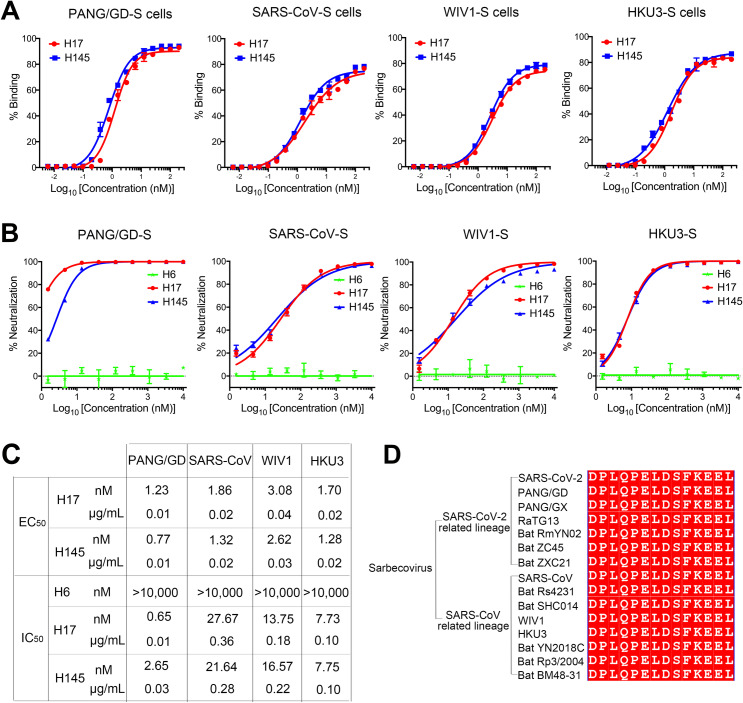
Cross-neutralizing capacity of H17 and H145 against other sarbecoviruses. (A) Binding capacity of H17 and H145 to sarbecoviral S proteins expressed on HEK-293T cells by flow cytometry. Data are means ± SD of duplicate samples. (B) Cross-neutralization activity of H17 and H145 against sarbecoviruses detected by pseudovirus neutralization assay. Data are means ± SD of triplicate samples. H6 was used as a negative control. (C) Table summarizing the binding EC50 value and the neutralizing IC50 value by flow cytometry and pseudovirus assay, respectively. (D) Epitope sequence alignment among sarbecoviruses, including SARS-CoV-2- and SARS-CoV-related lineages. Sequences were downloaded from the GISAID database and https://covariants.org.

SARS-CoV-2 and SARS-CoV are highly transmissible and pathogenic viruses within the sarbecovirus group. Other zoonotic sarbecoviruses, such as PANG/GD, WIV1, and HKU3, have also garnered significant attention due to their genomic similarities to SARS-CoV-2 and SARS-CoV as well as their potential to infect humans via cross-species transmission. To evaluate the inhibitory effects of H17 and H145 on these sarbecoviruses, we first investigated the binding capacity of H17 and H145 to sarbecoviral S proteins expressed on HEK-293T cells using flow cytometry. Both nanobodies showed strong binding to all the tested sarbecoviruses, with EC50 values ranging from 1.23 to 3.08 nM for H17 and 0.77 to 2.62 nM for H145, respectively (Fig 4A and 4C).

Next, we performed pseudovirus neutralization assays to assess the inhibitory effects of H17 and H145 against sarbecoviruses, including SARS-CoV, PANG/GD, WIV1, and HKU3. The results revealed potent cross-neutralization activity for both nanobodies, with IC50 values ranging from 0.65 to 27.67 nM for H17 and 2.65 to 21.64 nM for H145, respectively (Fig 4B and 4C). Furthermore, sequence alignment showed that the epitope sequence D_1139_PLQPELDSFKEEL_1152_ was highly conserved among representative sarbecoviruses, both in SARS-CoV-2-related lineage (SARS-CoV-2, PANG/GD, PANG/GX, RaTG13, RmYN02, ZC45, and ZXC21) and SARS-CoV-related lineage (SARS-CoV, Rs4231, SHC014, WIV1, HKU3, YN2018C, Rp3, and BM48–31) (Fig 4D).

### Neutralization mechanism of H17 and H145

To elucidate the neutralization mechanism of the two nanobodies, we first prepared the complex proteins of the individual nanobody in complex with the epitope SH-peptide (spanning residues D1139-D1153) and then resorted to crystallography for structure determination. We finally successfully crystallized the H145/SH-peptide complex, and its crystal structure was successfully resolved at 1.6-Å resolution. The final structure was refined to *R*_work_ = 0.1911 and *R*_free_ = 0.2020, respectively (S1 Table).

**Fig 5:**
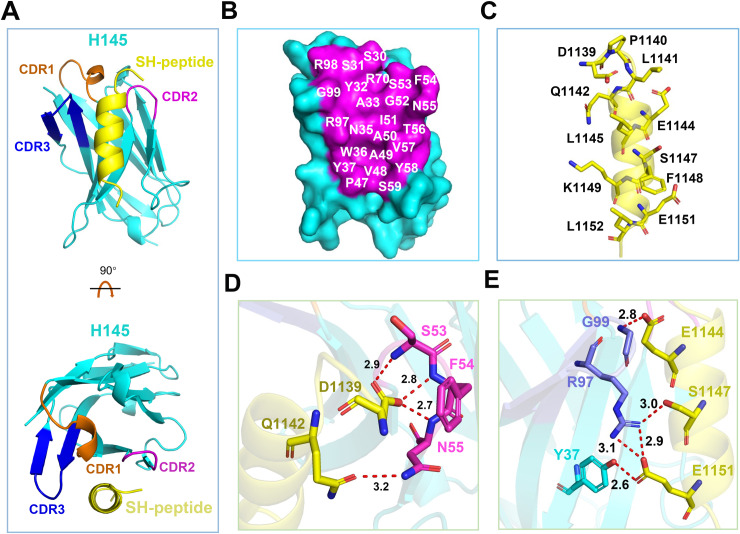
Complex structure of H145 bound to the epitope SH-peptide. (A) Cartoon representation of the H145/SH-peptide complex structure. Side view (upper) and top view (lower) of the complex structure are presented. H145 is in cyan, and SH-peptide in yellow. CDRs of H145 are shown in orange, magenta, and blue, respectively. (B-C) Residues involved in paratope recognition (the distance cutoff is 4.5 Å). Amino acids of H145 (B) and SH-peptide (C) involved in the contacts are shown. H145 is present in surface and the involed residues are coloured in magenta. SH-peptide is present in sticks and coloured in yellow. (D-E) Hydrogen-bond and salt-bridge interactions between H145 and SH-peptide (distance cutoff is 3.2 Å). Structures are presented and shown in cartoon and sticks. H145 is shown in cyan, and SH-peptide in yellow. CDRs of H145 are shown in orange, magenta, and slate, respectively.

In the structure, the H145 amino acids from Q1 to S114 and the SH-peptide residues from D1139 to D1153 were clearly traced in the electron-density map. As expected, the nanobody mainly used its three CDRs for epitope recognition (Fig 5A). These include S30-A33 in CDR1, I51-T56 in CDR2, and R97-G99 in CDR3. In addition, the paratope also involves several amino acids in the framework region, including N35-Y37, P47-A50, V57-S59, R70. These residues directly interact with SH amino acids D1139-Q1142, E1144-L1145, S1147-K1149, and E1151-L1152 (Fig 5B and 5C). Of note, amino acids D1139, Q1142, E1144, S1147, and E1151 in SH play a critical role in the epitope recognition by H145. These residues were observed to form a series of hydrogen-bonds and salt-bridges, by interacting with Y37, S53, F54, N55, R97, and G99 in the nanobody, to further stabilize the H145/SH-peptide binding (Fig 5D and 5E). These structural observations coincided well with our epitope mapping results, which showed that D1139 was indispensable in nanobody recognition.

We further analyzed the interface residues in the H145/SH-peptide complex structure using PDBePISA [34]. The results indicated that D1153 did not contribute to the buried surface area (BSA), whereas both D1139 and L1152 were involved in the interaction, with BSA values of 74.86 Å² and 70.92 Å², respectively (S2 Table). This suggests that both D1139 and L1152 participate in the epitope interaction, supporting our epitope-mapping studies confining the shortest epitope for H145 to S2 (D1139-L1152).

In comparison to H145, H17 differs, in sequence, by six amino acids (E1Q, V4L, A23E, V93I, Y103T, and P114S) (S7A Fig). These six residues, however, are far away from the epitope/paratope interface and would not be involved in epitope interaction (S7B Fig). Apparently, H17 and H145, while recognizing the same linear epitope sequence, possess exactly the same paratope residues. Therefore, the two nanobodies should share the same epitope binding mode.

**Fig 6:**
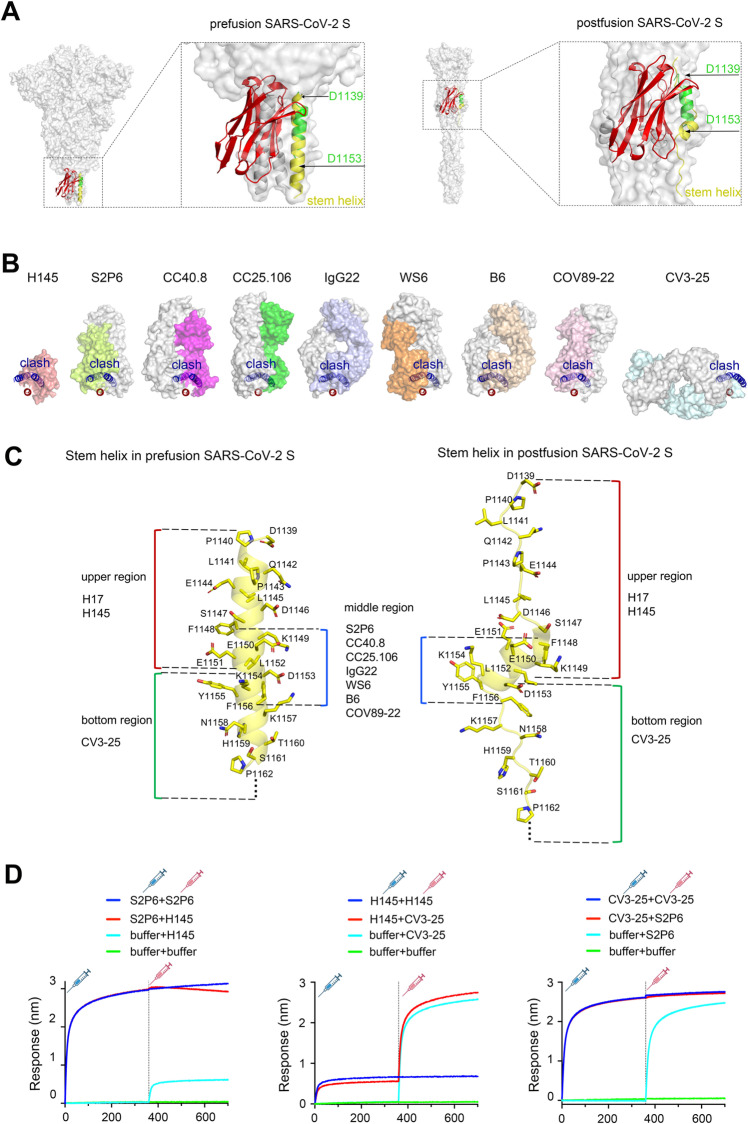
Structural basis for nanobody neutralization. (A) H145/SH-peptide complex structure superimposed on the reported SARS-CoV-2 spike trimer structures in both the prefusion (PDB: 6XR8) and the postfusion (PDB: 6XRA) states. Superimposition was done by aligning the complex of H145 (red) and SH-peptide (green) with the corresponding part in one of spike protomers (SH shown as yellow). (B) Binding modes of anti-SH nNbs and nAbs onto the stem helix (D1139-P1162) trimer structure in the SARS-CoV-2 prefusion state (PDB: 6XR8). The stem helix is shown in cartoon representation. One stem helix binding by nanobodies or antibodies is colored in red, and the other two, which clash with anti-SH nNbs or nAbs, are colored in blue. The light chain of each Fab of nAb is colored gray, and the heavy chain is in a different color for distinction. The Fab of nAbs is shown in surface. (C) Epitopes recognized by the two nanobodies and the reported anti-SH nAbs labeled on the SARS-CoV-2 stem helix (D1139-P1162, yellow) sturctures both in the prefusion (PDB: 6XRA) and postfusion (PDB: 6XR8) states. (D) Real-time BLI binding profiles of H145 and S2P6 (left), H145 and CV3-25 (middle), and S2P6 and CV3-25 (right) to immobilized S2 (T1076-Q1208) protein. The syringe icon in the figure was created using BioRender.com.

To structurally explore the inhibition mechanism for the two nanobodies, we compared our structure with the reported spike-trimer structures in both the prefusion (PDB: 6XR8) and the postfusion (PDB: 6XRA) states (Fig 6A). Structural superimposition clearly showed that the nanobody bound to the buried inner interface of the helical SH bundle. Such binding mode is incompatible with either the prefusion or the postfusion spike-trimer architecture, resulting in strong steric clashes with the neighboring protomers. In addition to the H17 and H145 nanobodies identified in this study, other thus-far reported anti-SH nAbs, such as S2P6 [23], CC25.106 [24], CC40.8 [35], IgG22 [36], B6 [37], WS6 [38], and COV89–22 [39], all target the unexposed SH inner face rather than the exposed helical region (Fig 6B), indicating a shared mechanism for entry inhibition by these anti-SH inhibitors. It is also notable that most of the reported anti-SH nAbs (e.g., S2P6, CCP40.8, and CC25.106) recognize an epitope in the middle region of SH (mainly spanning residues F_1148_KEELDKYF_1156_). Our nanobodies, however, recognize residues D1139-L1152 that are located in the upper region of SH. Another nAb, CV3–25 [40], recognizes an epitope spanning D1153-V1164 in the lower region of SH (Fig 6C). While the middle region remains predominantly helical in both the prefusion and postfusion states, the upper and lower regions would refold, along with the prefusion-to-postfusion conformational change in S, from a helical structure into an extended loop structure (Fig 6C). The binding of H17/H145 and CV3–25 to SH, therefore, should also inhibit the helix-loop transitions of the SH upper and lower regions, further blocking subsequent fusions between viral envelope and cell membrane.

A recent report has provided direct observations that antibodies targeting both the buried and the exposed surfaces of SH will impede the refolding of S2 [41]. This occurs by binding to a disrupted three-helix bundle and thus sterically blocking the back-zippering of HR2 onto the extended HR1 domains during the transition from the pre-hairpin intermediate to the postfusion state. In line with these findings, the binding of H145 and H17 to the viral spike protein is expected to cause steric clashes with adjacent protomers, thereby hindering the assembly of the S2 fusion machinery and blocking membrane fusion. It is noteworthy that neutralization via binding to the SH bundle appears to represent a common taking-action pattern for anti-SH nAbs.

We also investigated the potential compatibilities among nNbs/nAbs targeting different regions of SH. The upper-region targeting (as represented by H145), middle-region targeting (as represented by S2P6) [23], and lower-region targeting neutralizers (as represented by CV3–25) [40] were selected for structural and functional analyses. Initial alignments of the structures, including the H145/SH-peptide structure (PDB: 9LDS reported in this study), the S2P6 Fab/SH-peptide structure (PDB: 7RNJ), and the CV3–25 Fab/SH-peptide structure (PDB: 7NAB), revealed that H145 and CV3–25 could simultaneously bind to the stem helix, whereas S2P6 showed steric clashes with both H145 and CV3–25 (S8 Fig). To validate these observations, we performed competitive binding assays using BLI with immobilized S2 (T1076-Q1208). Echoing the structural observations, H145 is compatible with CV3–25, whereas S2P6 is incompatible with (competing against) both H145 and CV3–25 (Figs S9 and 6D). Our results suggest that H145 and CV3–25 may serve as a promising cocktail combination to synergistically enhance neutralization against SARS-CoV-2 and its variants.

### H17 and H145 bind to viral S in an acidification-insensitive manner

**Fig 7:**
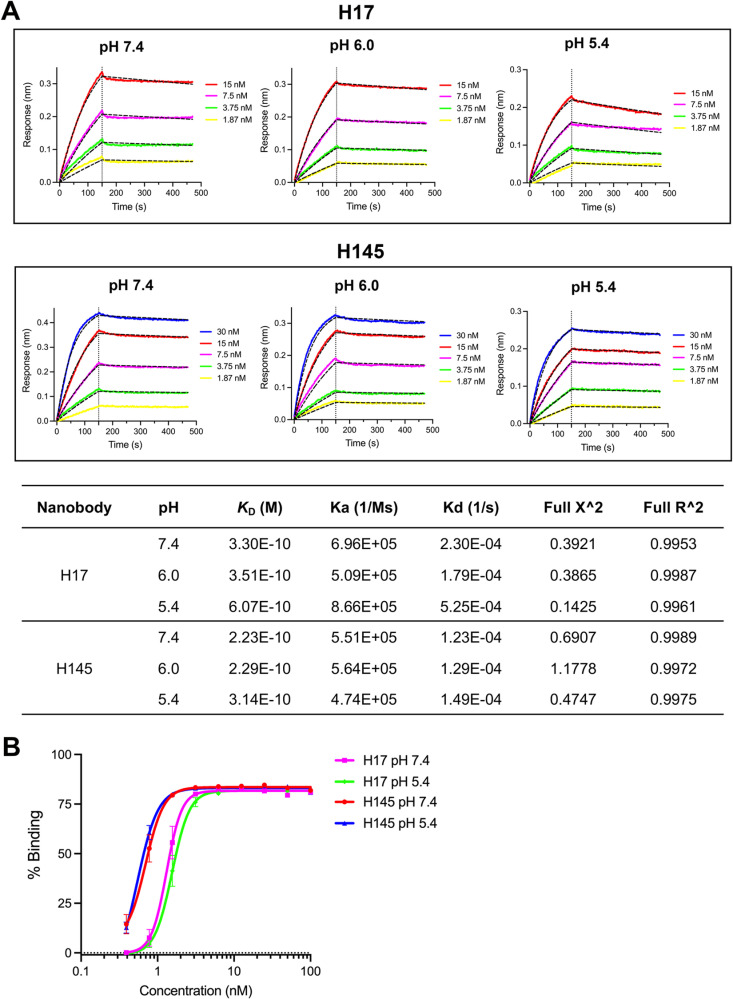
H17 and H145 bind to viral S in an acidification-insensitive manner. (A) S2 (T1076-Q1208) protein was biotinylated and immobilized. Slow-on/slow-off kinetic data are analyzed by the 1:1 binding model. Recorded binding profiles and calculated kinetic parameters are shown. (B) Binding capacity of the indicated nanobodies to SARS-CoV-2 spike on membrane of HEK-293T cells at different pH conditions (7.4 or 5.4). The percentage of H145 positive cells was detected by flow cytometry.

SARS-CoV-2 enters host cells via two distinct pathways: plasma membrane fusion at neutral pH (~7.5) or fusion within endosome at acidic pH (<6.0). Acidic pH could influence protein-protein interactions, including the antigen/antibody binding. Consequently, the acidic conditions within the endosome might hinder the attachment of S-targeting antibodies to viral S, therefore reducing their efficacy when the virus enters through the endosomal pathway. To investigate this, we explored the impact of acidic pH on the binding between our nanobodies and viral S. BLI results showed that the binding affinity of H17 to S2 (T1076-Q1208) was 0.330 nM, 0.351 nM, and 0.607 nM at pH-7.4, -6.0, and -5.4, respectively. For H145, the binding affinity was 0.223 nM, 0.229 nM, and 0.314 nM at pH-7.4, -6.0, and -5.4, respectively (Fig 7A). Since pH might influence the conformation of the spike protein, we further evaluated the binding capabilities of H17 and H145 to the spike protein on cell membranes under different pH conditions. The flow cytometry results revealed that our nanobodies maintained similar binding capacities to the spike protein at both pH 7.4 and 5.4 (Fig 7B). Thus, H17 and H145 bind to viral S in an acidification-insensitive manner, suggesting that their entry-inhibition activity would not be affected when SARS-CoV-2 enters via the endosomal pathway.

### Extended taking-action window of H17 and H145 for virus neutralization

**Fig 8:**
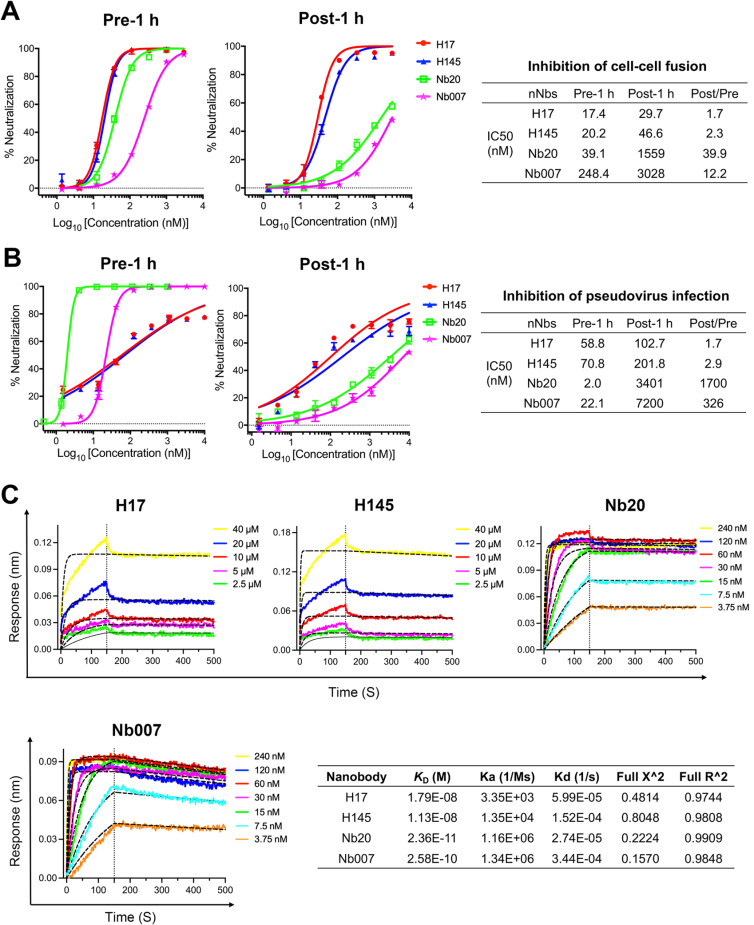
Extended taking-action window of H17 and H145 for virus neutralization. (A-B) Quantitative analysis of the inhibitory activity for anti-SH nNbs (H17 and H145) and anti-RBD nNbs (Nb20 and Nb007) by the syncytium-formation inhibition assay (A) and the pseudovirus neutralization assay (B). Time of adding nNbs was adjusted to two different time points: in the Pre-1 h group, nNbs were pre-incubated with HEK-293T/S cells or SARS-CoV-2 pseudovirus for 1 hour at 37°C and then mixed with HEK-293T-hACE2 cells, respectively; in the Post-1 h group, nNbs were added after HEK-293T/S and HEK-293T-hACE2 cells were pre-incubated for 1 h at 37°C or after SARS-CoV-2 pseudovirus and HEK-293T-hACE2 cells were pre-incubated for 1 h on ice, respectively. (C) Binding affinity of anti-SH and anti-RBD nNbs to the prefusion locked SARS-CoV-2 S trimer (F817P, A892P, A899P, A942P, K986P, V987P, R683A, and R685A) as the biotinylated and immobilized protein at pH 7.4 detected by BLI assay. Slow-on/slow-off kinetic data are analyzed by the 1:1 binding model. Recorded binding profiles and calculated kinetic parameters are shown.

To evaluate the taking-action window of our nanobidies for virus neutralization, we first employed the syncytium-formation inhibition assay by simulating two action modes. In the Pre-1 h group, nNbs were pre-incubated with HEK-293T/S cells for 1 hour before mixing with HEK-293T-hACE2 cells to mimic the action mode in which nNbs pre-bound to S before the virions’ attachment to plasma membrane. In the Post-1 h group, nNbs were added after co-culturing HEK-293T/S and HEK-293T-hACE2 cells for 1 hour to mimic the action mode in which nNbs act after the virions’ adherence to the target cell surface. In both action modes, the performance of anti-SH nNbs (H17 and H145) was compared head-to-head with reported anti-RBD nNbs (Nb007 [28] and Nb20 [29]). The results showed that both anti-SH and anti-RBD nNbs effectively blocked S-mediated cell-cell fusion in the Pre-action mode, with IC50 values of 17.4 nM and 20.2 nM for H17 and H145 and 39.1 nM and 248.4 nM for Nb20 and Nb007, respectively. In the Post-action mode, the inhibition efficacy of H17 and H145 was slightly reduced, with IC50 values of 29.7 nM (decreased by ~1.7-fold) and 46.6 nM (decreased by ~2.3-fold), respectively. However, the inhibitory activity of Nb20 and Nb007 decreased significantly. The IC50 values were determined to be 1559 nM and 3028 nM, representing ~39.9- and ~12.2-fold decrease in efficacy, respectively (Fig 8A).

We also performed the pseudovirus neutralization assay in the similar Pre- and Post-action modes. In the former experiment, nanobodies were pre-incubated with SARS-CoV-2 pseudovirus. This allows the nanobodies to pre-bind viral S before the virions attach to plasma membrane. As expected, both anti-SH and anti-RBD nNbs effectively neutralized the viral infection. The IC50 values were determined to be 58.8 nM and 70.8 nM for H17 and H145 and 2.0 nM and 22.1 nM for Nb20 and Nb007, respectively. In the latter experiment, SARS-CoV-2 pseudovirus was pre-incubated with HEK-293T-hACE2 cells on ice to allow viral adherence to cell surface. The nanobodies were then added for virus neutralization. While anti-SH nNbs still effectively neutralized SARS-CoV-2 infection (with IC50 values of 102.7 nM for H17 and 201.8 nM for H145 which represents ~1.7- and ~2.9-fold decrease in efficacy), the neutralizing activity of anti-RBD nNbs decreased by ~1700- and ~326-fold, respectively (with IC50 values of 3401 nM for Nb20 and 7200 nM for Nb007) (Fig 8B).

We further compared the binding affinity of these tested anti-SH and anti-RBD nNbs to the prefusion locked S trimer in the BLI experiment. The results showed that the binding affinity of H17 and H145 to prefusion S was 17.9 nM and 11.3 nM, respectively (Fig 8C). The binding affinity of Nb20 and Nb007 to prefusion S, however, was determined to be 0.02 nM and 0.25 nM, respectively (Fig 8C). The anti-RBD nNbs, therefore, were much better S-binders than the anti-SH nNbs. However, the anti-SH nNbs were much more effective in the Post-action mode. These results indicate that H17 and H145, which target the S2 fusion core, possess a better taking-action window for virus neutralization when compared to Nb007 and Nb20 that neutralize by competing against ACE2 binding to RBD.

### *In-vivo* protection of SARS-CoV-2 infection in mice

To evaluate the protective efficacy of our nanobodies against SARS-CoV-2 infection, we further performed the *in-vivo* tests using the pseudovirus following the intrathoracic virus challenge route as previously reported [42,43]. The study was carried out in BALB/c mice with Omicron pseudovirus as prior studies have shown that the N501Y mutation in spike could enhance mouse ACE2 binding, facilitating cross-species transmission of SARS-CoV-2 in mice [44–46]. The lung tissues were harvested 3 days post-challenge, and the percentage of the virus-infected cells was quantified using flow cytometry. As shown in S10 Fig, prophylactic intranasal administration of nanobody H145 (10 mg/kg) resulted in a significant reduction in the virus-infected cells in the lungs compared to the control group treated with PBS. These findings suggest that our nanobodies could provide in-vivo protection against SARS-CoV-2. While nanobodies have been shown to be suitable for inhalation-based delivery for pulmonary infectious diseases [47,48], we believe that our nanobodies hold clinical potential as inhalable therapeutics for preventing SARS-CoV-2 infection.

## Discussion

Despite of global efforts, COVID-19 remains a worldwide public health crisis. Several monoclonal antibodies targeting the S1-RBD have been approved for the prevention and/or treatment of COVID-19, such as Tixagevimab/Cilgavimab [49] and Bebtelovimab [50]. However, the newly-emerged Omicron variants, particularly XBB, BA.2.75, and BA.2.86, possess over 20 mutants in S1-RBD which grant them enhanced transmissibility and significant immune evasion capacity, markedly compromising the efficacy of most approved antibodies. Compared to S1-RBD, which is prone to mutational escape, the S2 subunit is a better target for the development of broad-spectrum anti-SARS-CoV-2 drugs. Several well-conserved elements in S2, including the fusion peptide (FP), the SH, and the HR1/HR2 bundle, have been targeted for drug design [19,51]. Targeting FP, several studies have reported that anti-FP antibodies (e.g., COV44–62, COV44–79, VN01H1, VP12E7, C77G12, 76E1, and fp.006) possess broad neutralizing activity and are resistant to viral escape by forming steric hinderance to prevent S2′ cleavage and/or inhibiting FP insertion into the host cell membrane [20,52–54]. Targeting HR1, the HR2-based peptides are shown to bind to the HR1 groove, thus blocking viral 6HB formation and subsequent membrane fusion [22,55,56]. Two nasal sprays based on HR2-derived peptides, namely HY3000 [55] and EK1 [56], are undergoing clinical trials for the prevention or treatment of COVID-19 (*ChiCTR2200065930, CTR20230011*). Targeting SH, the H17 and H145 nanobodies reported in this study, as well as the anti-SH antibodies identified previously (e.g., S2P6 [23], CC25.106 [24], CC40.8 [35], IgG22 [36], B6 [37], WS6 [38], COV89–22 [39], CV3–25 [40], and hr2.016 [54]), all exhibit broad-spectrum neutralizing activity by disrupting the SH bundle and sterically blocking fusogenic rearrangement of S2. Targeting HR2, a five helix-bundle (5HB) design can interact specifically with viral HR2, disturbing the HR1/HR2 bundle formation and blocking membrane fusion [57]. All these S2-targting inhibitors are able to neutralize multiple SARS-CoV-2 variants and even cross-neutralize a series of sarbecoviruses. Notably, the FP epitope and the HR1 groove are buried and inaccessible in prefusion S, requiring a conformational change to enable these nAbs to bind to FP or HR1. In contrast, the anti-SH nNbs/nAbs can pre-bind to viral S, thus providing a longer binding-window than anti-FP nAbs and HR2-derived peptides.

It is also notable that the anti-SH nNbs/nAbs also show superior taking-action window to the anti-RBD nNbs/nAbs. When the nanobodies have sufficient time to pre-bind to the virion as mimicked in our Pre-action mode, both anti-RBD and anti-SH nNbs can effectively prevent virus infection. When the virion already engages ACE2 as mimicked in our Post-action mode, however, the dissociation of S1 would cause anti-RBD nNbs lose the binding capacity and therefore the inhibition efficacy. In contrast, anti-SH nNbs can still bind to the S2 subunit, hindering S2-induced membrane fusion and interfering with virus infection. Accordingly, the anti-SH nNbs H17 and H145 still show effective inhibition when SARS-CoV-2 pseudovirus is pre-incubated with HEK-293T-hACE2 cells. The neutralizing activity of anti-RBD nNbs, however, are significantly compromised. These findings suggest that the anti-SH nNbs/nAbs would still be effective after the virions adhere to the cell surface, superior to anti-RBD nNbs/nAbs that take action mainly prior to the viral binding to target cells. It should also be noted that the anti-SH nNbs are more effective in inhibiting cell-cell fusion than virus-cell fusion. Conversely, anti-RBD nanobodies exhibit approximately tenfold higher efficacy in pre-attachment virus-cell fusion assays but are less effective in preventing cell-cell fusion. These observations might suggest that anti-SH nNbs bind slowly, working better with slow fusion processes, indicating potential limitations in their efficacy against viruses that spread via a fast fusion process.

When SARS-CoV-2 enters via the endosomal pathway, the proteolytic cleavage of viral S would be mediated by lysosomal cathepsins, thereby leading to membrane fusion at acidic pH [58]. During this process, decreased pH might disrupt antigen-antibody interactions. For instance, S2P6, an anti-SH nAb, binds to viral S with an affinity that is ~6-fold lower at endosomal pH than at serological pH [23]. For the anti-RBD antibody CR3022, a low-pH-induced affinity drop can be as much as 1000-fold [59]. In contrast, we show that the binding of H17 and H145 to SH are almost not affected by acidification. The two nanobodies should therefore inhibit the viral infection with similar efficacy when fusion occurs either on the cell surface or within the endosomes.

By targeting S2, our nanobodies are expected to inhibit the virus entry by blocking membrane fusion, which is a complicated process that will be affected by the intrinsic properties of the S protein. In comparison to the pre-Omicron variants, including Delta, Beta, Kappa, Mu, and etc, the Omicron variants have accumulated approximately 30 additional mutations in S. These amino acid substitutions have been shown to lead to changes in the properties of S (e.g., Stability [60], structure [61], receptor binding capacity [62], S1/S2 dissociation [63]), which will in turn affect the neutralization efficacies of the antibodies targeting S2. Although the epitope sequence of S2 (D1139–L1152) recognized by our nanobodies is highly conserved among SARS-CoV-2 variants and even across sarbecoviruses, their entry inhibition efficiencies are shown to be more potent against Omicron than against the earlier pre-Omicron variants. Similar phenomena have also been observed for other S2-targting neutralizers [57,64].

Finally, it is interesting that H8, H23 and H88 nanobodies show strong signals in the ELISA assay but are negative in the flow cytometry assay. This suggests that they bind only to soluble S2 but not spike on the membrane. Identifying the epitopes for these nanobodies would be valuable for further characterizing this highly conserved S2 region.

Taken together, we believe the two nanobodies identified in this study, featured with a broad-spectrum neutralizing activity, an extended taking-action window and an acidification-insensitive binding to viral S, represent promising entry inhibitors against SARS-CoV-2.

## Materials and methods

### Alpaca immunization and S2-specific phage clone screening

The SARS-CoV-2 S2-specific nanobody repertoire was derived from CHENGDU NB BIOLAB CO., LTD, as previously described [27]. We immunized an alpaca with a trimeric S2 fusion-core-based antigen, namely S2 (T1076-Q1208), to prepare the S2-specific VHH phage library. Briefly, one alpaca was immunized with 0.5 mg antigen by subcutaneous injection on day 0 and then boosted with 0.25 mg antigen on days 21 and 42. Peripheral blood mononuclear cells (PBMCs) were isolated from the blood on day 49. Total RNA was then extracted from PBMCs and reverse-transcribed into cDNA. After nested PCR amplification, the VHH genes were separated and inserted into the pComb3XSS vector. The ligated vectors were electroporated into TG1-competent cells to prepare the phage libraries. Following a two-round selection process, individual phage clones were rescued and subjected to testing for their initial binding ability to the S2 protein, screening for S2-specific phage clones.

### Expression and purification of antigen-specific nanobodies

SARS-CoV-2 S2 (T1076-Q1208) antigen-specific nanobodies were produced as previously described [28]. Briefly, the coding sequence for each nanobody with a C-terminal 6×His tag was cloned into the expression vector pNCMO2, and subsequently transformed into Brevibacillus choshinensis SP3 cells. Then, they were grown in MTN medium at 37°C for one day and then at 30°C for four days for nanobody expression. The supernatant was resuspended in PBS buffer for 2 hours and then centrifuged at 9,000g for 40 minutes to septate cells. Following incubation with Ni-TED NUPharose FF beads (NUPTEC), the supernatant underwent gel filtration using a Superdex 75 Increase 10/300 GL column (GE Healthcare) in HEPES buffer (20 mM HEPES, pH 7.4, 150 mM NaCl). The protein was concentrated with a 3 kDa MWCO centrifugal filter unit (Merck Millipore). The UV absorbance curves were recorded at 280 nm, and the purification of nanobodies was determined by polyacrylamide gel electrophoresis.

### Expression and purification of S2-associated truncated proteins

To prepare the SARS-CoV-2 S2 (T1076-Q1208, GenBank: MN908947.3) antigen protein, a GST tag at the N terminus and a T4 fibritin trimer domain with a 6×His tag at the C terminus were added for protein secretion, purification, and folding into a trimer. The coding sequence named S2“ (T1076-Q1208) was cloned into the pET-21a expression vector, transformed into BL21 (DE3) *E.coli* (Beijing Tsingke Biotech Co., Ltd.), grown in Luria-Bertani (LB) at 37°C until OD 0.8, followed by gene induction using 1 mM IPTG for 16 hours at 16°C. Subsequently, *E. coli* were harvested, resuspended, lysed by a high-pressure homogenizer, and then recollected via centrifugation to separate cell debris. The lysate was incubated with Ni-NTA resin (QIAGEN) for 2 hours with rotation at 4°C. The resin was then washed with HEPES buffer, wash buffer (HEPES buffer with 10 mM imidazole), and then elution buffer (HEPES buffer with 500 mM imidazole). A 10 kDa MWCO centrifugal filter unit (Merck Millipore) was used to concentrate the eluted protein before it was injected onto a Superdex 200 Increase 10/300 GL column (GE Healthcare) for gel filtration. The UV absorbance curves were recorded at 280 nm, and polyacrylamide gel electrophoresis was used to determine the protein purification. The S2” (T1076-Q1208) protein was digested with PSP protease to remove GST. Further protein purification was conducted with GST resin (Smart-Lifesciences) and then flowed through onto a Superdex 200 Increase 10/300 GL column (GE Healthcare) in HEPES buffer (20 mM HEPES, pH 7.4, 150 mM NaCl) to obtain the S2 (T1076-Q1208) antigen.

For functional experiments, we designed a series of truncated S2 proteins, fused with GST at the N-terminus. The coding sequences were cloned into an expression vector. These coding sequences encompass S2 amino acids spanning T1076-I1183, T1076-H1159, T1076-N1135, T1076-E1111, T1100-N1135, G1124-H1159, F1148-I1183, D1168-Q1208, V1133-S1147, T1136-E1150, D1139-D1153, Q1142-F1156, L1145-H1159, P1140-D1153, D1139-L1152, P1140-L1152, L1141-L1152, D1139-E1151, and D1139-E1150. The expression and purification of these S2-associated truncated proteins were conducted using the same protocols as previously described.

### Crystallization

For crystallization screening, the nanobody/SH-peptide (H145/SH-peptide or H17/SH-peptide) complex was prepared. Briefly, homologous nanobodies were mixed with SH-peptide in a 1:3 molar ratio and incubated on ice for 2 hours. The final concentrations of the complexes were adjust to 10 mg/mL or 20 mg/mL. Crystallization screens were set up in 96-well plates using the sitting-drop vapor diffusion method with commercial crystallization kits (Hampton Research and Molecular Dimensions). Specifically, 1 μL of the nanobody/SH-peptide complex was mixed with 1 μL of the reservoir solution and equilibrated against 75 μL of the reservoir solution at 18°C.

### Data collection and structure determination

For data collection, the crystals were cryoprotected by quick-dipping in reservoir solution supplemented with 20% (v/v) glycerol and flash-cooled in a cryogenic nitrogen stream. X-ray diffraction data were collected at the Shanghai Synchrotron Radiation Facility (SSRF) beamline BL18U1 [65]. A single crystal of the H145/SH-peptide complex, grown in 0.1 M HEPES pH 7.5, 70% v/v (+/-)-2-Methyl-2,4-pentanediol, diffracted to 1.6–1.8 Å. Indexing, integration, and scaling were processed using HKL3000 [66]. The structure was solved by molecular replacement using PHASER [67] from the CCP4 [68] suite, with the nanobody structure (PDB: 5G5R) as the search model. Model building was completed with COOT [69] and refined with PHENIX [70]. The stereochemical quality of the final models was assessed using PROCHECK [71]. Final data processing and structure refinement statistics are listed in S1 Table. All structural figures were generated using PyMOL (http://www.pymol.org).

### Flow cytometry assay

The binding of nanobodies to cell surface-expressed spike proteins was detected by flow cytometry. Plasmids encoding the spike proteins of various coronaviruses, including SARS-CoV-2 (MN908947.3), SARS-CoV (AY278554.2), PANG/GD (QLR06867.1), HKU3 (QND76034.1), and WIV1 (AGZ48828.1), in the pCAGGS vector, were transfected into HEK-293T cells using the Lipo8000 transfection reagent (Beyotime) according to the manufacturer’s protocol. After 40 hours, 500,000 HEK-293T cells were washed and incubated with His tag nanobodies for 1.5 hours. To assess membrane-anchored spike binding under physiological (pH 7.4) and endosomal (pH 5.4) conditions, a truncated 10-min incubation protocol was implemented to mitigate low-pH-induced cellular perturbations. After removing unbound nanobodies by centrifugation, the cells were labeled with PE-conjugated anti-His antibody (Miltenyi Biotec, #130-120-718) and then were counterstained with LIVE/DEAD Near-IR Viability kit at 4°C for 20 minutes. The cells were washed with PBS to remove non-specific binding antibodies. A flow cytometer (ACEA NovoCyte) was then used to detect PE-positive cells. All data were analyzed using Flowjo 10 software.

### Enzyme-linked immunosorbent assay (ELISA)

96-well microtiter plates were coated with S2 (T1076-Q1208) protein or a series of truncated S2 proteins at 200 ng/well in a solution of 0.05 M carbonate-bicarbonate pH 9.6 at 4°C overnight. These proteins were devoid of the His tag. To identify S2-binding nanobodies, twenty nanobodies at concentrations of 200 nM or 1,000 nM were added to S2 (T1076-Q1208) protein-coated plates for 1.5 hours at room temperature following the standard washing and blocking steps. Five-fold serial dilutions of purified nanobodies (H17 and H145), ranging from 200 nM to 0.064 nM, were added to the truncated S2 protein-coated plates to identify the epitopes of H17 and H145, incubating for 1.5 hours at room temperature. HRP-conjugated secondary antibodies against the His-tag (Merck Millipore) were diluted 1:5000 and incubated with the wells for 1 hour. In each step, the wells were thoroughly washed with PBST (PBS, 0.1% Tween 20). Finally, TMB solution (Beyotime) was added to react with the HRP conjugates at room temperature. The reaction was stopped using 2 M HCl. Absorbance was measured at 450 nm using a microplate reader (BioTek).

### Bio-layer interferometry (BLI) assay

The BLI assay was conducted using an Octet Red 96 System (ForteBio). Initially, BLI was utilized to detect the binding ability of H17 and H145 to the truncated S2 proteins to identify the shortest epitopes that the nanobodies can recognize. Briefly, nanobodies (H17 and H145) were biotinylated using the Biotinylation Kit (BMD Lab Service, G-MM-IGT) according to the manufacturer’s instructions. They were then immobilized at 10 μg/ml on pre-wet streptavidin (SA) biosensors to achieve a maximum response of approximately 2 nm. Subsequently, 200 nM truncated S2 proteins were flowed over the captured nanobody surface for association, followed by dissociation with buffer. The assay was performed using HEPES buffer (20 mM HEPES pH 7.4, 150 mM NaCl, 0.05% (v/v) Tween-20).

The binding affinity of H17 and H145 to S2 (T1076-Q1208) was also determined by BLI at pH 7.4, 6.0, and 5.4, respectively. In brief, S2 (T1076-Q1208) protein was biotinylated with the Biotinylation Kit and immobilized at 10 μg/ml on pre-wet SA biosensors to achieve a maximum response of approximately 2 nm. Two-fold serial dilutions of purified nanobodies, ranging from 15 nM to 1.87 nM for H17 or from 30 nM to 1.87 nM for H145, were flowed over the captured S2 surface for association and then dissociation with buffer. The assay was conducted using various buffers, including 20 mM HEPES pH 7.4, 20 mM MES pH 6.0, or 20 mM MES pH 5.4, along with 150 mM NaCl and 0.05% (v/v) Tween-20. For the competitive binding assay, SA-immobilized S2 (T1076-Q1208) protein was prepared following the protocol established for kinetic assays. The S2P6 and CV3–25 antibodies were expressed and purified as previously reported [23,40]. For sequential epitope competition, the biosensor was first exposed to 200 nM primary antibody for 350 s, followed by immediate injection of 200 nM secondary antibody for an additional 350 s without dissociation monitoring between steps.

The biotinylated SARS-CoV-2 S protein, a super stable trimer (Cat. No. SPN-C82E9 from ACROBiosystems), is the ectodomain of the SARS-CoV-2 S protein (V16-P1213). It contains a T4 fibritin trimerization motif and a His tag at the C-terminus. Proline and alanine substitutions (F817P, A892P, A899P, A942P, K986P, V987P, R683A, and R685A) are introduced to stabilize the SARS-CoV-2 S protein in a trimeric prefusion state and remove the furin cleavage site. The reconstituted protein at 10 μg/ml was loaded onto pre-wet SA biosensors to achieve a maximum response of approximately 2 nm. Two-fold serial dilutions of purified nanobodies, ranging from 40 nM to 2.5 nM (for H17 and H145) or from 240 nM to 3.75 nM (for Nb20 and Nb007), were flowed over the captured S surface for 150 seconds of association, followed by 350 seconds of dissociation with buffer. The assay was conducted using HEPES buffer (20 mM HEPES pH 7.4, 150 mM NaCl, 0.05% (v/v) Tween-20). All BLI data analysis was performed using ForteBio Data Analysis 12.0 software with a 1:1 binding mode.

### Western blot (WB) assay

The truncated S2 proteins (without His tag) were prepared and separated on an SDS-PAGE gel by electrophoresis, followed by transfer onto polyvinylidene fluoride (PVDF) membranes. The membranes were blocked with 5% non-fat milk in 20 mM Tris, pH 7.5, 150 mM NaCl, and 0.1% Tween-20 for 2 hours at room temperature. Subsequently, H17 and H145 nanobodies (with His tag) at 1 μg/ml were added and incubated with the membrane for 1 hour at room temperature. After washing, HRP-conjugated secondary antibodies against the His-tag (Merck Millipore) were diluted 1:8000 and incubated with the membranes for 1 hour at room temperature. Finally, the bands were washed and detected using an enhanced chemiluminescence system (Fusion Solo 6S Edge, VILBER).

### Syncytium-formation inhibition assay

The SARS-CoV-2 S-mediated cell-cell fusion assay, also known as the syncytium-formation inhibition assay, was conducted as previously described [72]. In brief, HEK-293T cells were co-transfected with the plasmids pCAGGS-SARS-CoV-2 S and pCAGGS-EGFP using the Lipo8000 transfection reagent (Beyotime), referred to as HEK-293T/S/EGFP cells. HEK-293T cells transfected solely with the plasmid pCAGGS-EGFP served as the negative control cells, named HEK-293T/EGFP. ACE2 (GenBank: BAB40370.1) stable-expressing HEK-293T cells, referred to as HEK-293T-hACE2, were prepared as described in our previous report. First, 25,000 effector cells (HEK-293T/S/EGFP) were pre-incubated with nanobodies at the specified concentrations in an incubator for 1 hour. These mixtures were then added to 50,000 HEK-293T-hACE2 cells to facilitate cell-cell fusion. After a 6-hour incubation, three random fields in each well were imaged using a fluorescence microscope (Nikon).

For postfusion inhibition, 25,000 HEK-293T/S/EGFP cells were co-cultured with 50,000 HEK-293T-hACE2 cells for 1 hour in the incubator, followed by the addition of serially diluted nanobodies (H17, H145, Nb20, and Nb007) to simulate the action mode where inhibitors act after virions have adhered to the target cell surface. After 6 hour of incubation, three random fields in each well were imaged using a fluorescence microscope (Nikon).

ImageJ software (version 1.46) was employed to assess and quantify the area of fused and unfused cells. The inhibition activity of cell-cell fusion was calculated using the formula (P-E)/(P-N)×100%, as previously described. Here, “N,” “P,” and “E” represent the green areas of cell-cell fusion in the negative group (293T/S/EGFP cells with PBS), the positive group (293T/S/EGFP cells co-cultured with 293T-ACE2 cells), and the experimental groups (293T/S/EGFP cells co-cultured with 293T-ACE2 cells in the presence of nanobodies), respectively. The IC50 values were determined using GraphPad Prism 8 software. All experiments were performed on at least three biologically independent samples.

### Pseudovirus assays for nanobody neutralization

Pseudotyped viruses were produced as previously described [73]. Using the Lipo8000 transfection reagent (Beyotime), the S plasmid in the pCAGGS vector and an Env-defective luciferase-expressing HIV-1 plasmid in the pNL4–3.luc.RE vector were co-transferred into HEK-293T cells. S plasmids were derived from human and animal sarbecoviruses, including SARS-CoV-2 (MN908947.3), SARS-CoV (AY278554.2), PANG/GD (QLR06867.1), HKU3 (QND76034.1), and WIV1 (AGZ48828.1). After 48 hours of transfection, cell supernatants were centrifuged to remove cell debris, then concentrated to collect pseudovirus and stored at -80°C for subsequent use. Pseudoviruses for SARS-CoV-2 variants (BA.1, BA.2, BA.4/5, BA.2.12.1, BF.7, BA.2.75, XBB.1.5, XBB.1.16, JN.1, KP.3, P.1 501Y.V3, B.1.351, B.617.1, B.1.617.2, and B.1.621) were purchased from Genomeditech. The mutation sites of S variants are listed in S3 Table.

The pseudovirus neutralization assay was conducted as follows: 10,000 HEK-293T-hACE2 cells were cultured in each well of a 96-well plate for 6 hours. Serially diluted nanobodies were pre-incubated with titrated pseudovirus for 1 hour at 37°C. The mixtures were then added to HEK-293T-hACE2 cells. Following a 16-hour infection period, wells were replenished with fresh DMEM media containing 10% FBS for an additional 48 hours. This method is also applicable for prefusion inhibition of pseudovirus neutralization.

Postfusion inhibition of the pseudovirus neutralization assay was conducted as previously described [38]. Briefly, HEK-293T-hACE2 cells and titrated pseudovirus were kept on ice for 1 hour. The titrated pseudovirus was then incubated with HEK-293T-hACE2 cells on ice for an additional hour to allow the virus to attach to ACE2-expressing cells. After three washes, 10,000 cells were incubated with 3-fold diluted nanobodies on ice for additional hours before returning to the incubator for 16 hours. Wells were then replenished with fresh DMEM media containing 10% FBS for an additional 48 hours. Finally, luciferase activity was measured using the One-LumiTM II Firefly Luciferase Assay Kit according to the manufacturer’s instructions (Beyotime). All experiments were performed on at least three biologically independent samples.

### *In-vivo* evaluation of nanobody H145 against SARS-CoV-2 infection

Six-week-old female BALB/c mice (20~22 g, Beijing HFK Bioscience Co., Ltd.) were maintained under specific pathogen-free conditions at the State Key laboratory of Biotherapy, Sichuan University. All experimental protocols were conducted in a Biosafety level 2 facility in accordance with the Guide for the Care and Use of Laboratory Animals of Sichuan University, and the study was approved by the Institutional Animal Care and Use Committee of Sichuan University (Approval No. 20240305059). To assess the *in-vivo* protective efficacy of nanobody H145 against SARS-CoV-2 infection, nine BALB/c mice were randomly assigned to three experimental groups: negative control, positive control, and prophylactic groups. Mice in the positive control group were intrathoracically challenged with 25 μl of 4×10^8^ RLU SARS-CoV-2 BA.2 pseudotyped virus (GFP-Luciferase; Genomeditech, Shanghai, GM-0220PV86). In the prophylactic group, H145 (10 mg/kg) was administered intranasally 2 hours prior to challenge. The negative control group received only PBS intranasally without viral exposure. Three days post-infection, mice were euthanized, and lung tissues were harvested and homogenized using tissue lysis solution (Absin, abs9482). To quantify BA.2-infected cells, lung cells were labeled with the LIVE/DEAD Near IR Viability kit (Invitrogen) at 4°C for 20 minutes, followed by PBS washing. GFP-positive cells were quantified by flow cytometer (ACEA NovoCyte). All data were analyzed using Flowjo 10 software.

### Statistical analyses

All statistical analyses were carried out using GraphPad Prism 8.0 software. Detailed statistical methods are described in the method details or figure legends.

## Supporting information

S1 Fig Purification and gel-filtration characteristics of SARS-CoV-2 S2 (T1076-Q1208) protein. Solution behaviors of SARS-CoV-2 S2’‘ (T1076-Q1208) protein before (A) or after (B) PSP digestion on a Superdex 200 Increase 10/300 GL column. Inset figures show the SDS-PAGE analyses of the pooled samples. S2’‘ (T1076-Q1208) protein is fused with GST at the N terminus and T4 fibritin with His at the C terminus.
(TIF)

S2 Fig Amino-acid sequence alignment of the 20 nanobodies. The CDR regions are marked.
(TIF)

S3 Fig Solution behaviors of the 20 nanobodies on a Superdex 75 Increase 10/300 GL column. Inset figures show the SDS-PAGE analyses of the pooled samples.
(TIF)

S4 Fig Solution behaviors of the indicated truncated S2 proteins on a Superdex 200 Increase 10/300 GL column. Inset figures show the SDS-PAGE analyses of the pooled samples. These truncated proteins are fused with GST tag at the N terminus.
(TIF)

S5 Fig Identification epitope recognized by H17 and H145. (A) H17 and H145 recognize a linear epitope S2 (T1076-H1159) detected by WB. (B) Multi-concentration ELISA-binding profile of H17 or H145 to the indicated S2 antigen. OD450 emissions are plotted as curves. Data are means ± SD of triplicate samples. (C) Binding ability of the indicated S2 antigen to H17 or H145 analyzed by BLI. Immobilized biotinylated H17 or H145 was saturated in binding with the indicated S2 proteins. Single association and dissociation curves were detected. (D) Summary of the binding features of H17 and H145 to S2-related truncated proteins detected by WB, ELISA, and BLI. b., binding; n.b., no binding.
(TIF)

S6 Fig Sequence conservation logos of the amino acids spanning SARS-CoV-2 S2 stem-helix of N1135-V1164 (n=7107). The amino acids of the reported SARS-CoV-2 spike protein was retrieved from the NCBI database and removed erroneous sequences using MEGA7 software, resulting in a final dataset of 7,107 protein sequences. The sequence conservation of the total stem-helix amino acid sequence (N1135-V1164) was conducted using WebLogo (https://weblogo.threeplusone.com). The overall height of the stack indicates the sequence conservation at that position, while the height of symbols within the stack indicates the relative frequency of each amino acid at that position.
(TIF)

S7 Fig Amino-acid differences between H17 and H145. (A) Amino-acid sequence of H17 and H145. Different residues are shown in purple. CDR regions are marked. (B) Six residues in H145 that are different from H17 are shown in the crystal structure of the H145/SH-peptide complex in purple. H145 is shown as surface in aquamarine, and SH-peptide is shown as cartoon in yellow. Side view (left) and top view (right) of structures are presented and shown.
(TIF)

S8 Fig Alignments of the H145/SH-peptide structure, the S2P6 Fab/SH-peptide structure, and the CV3–25 Fab/SH-peptide structure. (A-C) Superimposed structures of three antibody fragment/SH-peptide complexes (surface representation) bound to the prefusion SARS-CoV-2 S-trimer (gray cartoon, PDB: 6XR8). (A) H145/SH-peptide complex (PDB: 9LDS reported in this study), colored red. (B) S2P6 Fab/SH-peptide complex (PDB: 7RNJ), colored lightblue. (C) CV3–25 Fab/SH-peptide complex (PDB: 7NAB), colored yellow. In all panels, the stem-helix of one S-trimer monomer is highlighted in cyan (residues D1139-P1162).
(TIF)

S9 Fig Solution behaviors of the anti-SH nAbs S2P6 and CV3–25 on a Superdex 200 Increase 10/300 GL column. Inset figures show the SDS-PAGE analyses of the pooled samples.
(TIF)

S10 Fig Prophylactic efficacy of nanobody H145 against SARS-CoV-2 infection. (A) Experimental design for evaluating H145 prophylaxis. Six-week-old female BALB/c mice received intranasal administration of H145 (10 mg/kg) or PBS 2 hours prior to intrathoracic challenge with 4×10^8^ relative light unit (RLU) SARS-CoV-2 Omicoron pseudotyped virus (GFP-Luciferase). Negative controls received PBS without viral challenge. This figure was created using BioRender.com. (B) Virus-infected cells in lung tissues at 3 days post-challenge, quantified by flow cytometry analysis of tissue homogenates. Data are presented as means ± SEM. Statistical significance was determined by unpaired t-test (**P<0.01, ***P<0.001).
(TIF)

S1 Table Data collection and structure refinement statistics. In each case, a single crystal was used to collect the data. Values in parentheses are for the highest-resolution shell.
(DOCX)

S2 Table The interface residues in the H145/SH-peptide complex. Surface area of H145 on the SARS-CoV-2 S2 stem-helix is shown. The buried areas were calculated with Proteins, Interfaces, Structures and Assemblies (PDBePISA). HSDC (Residues making **H**ydrogen/**D**isulphide bond, **S**alt bridge or **C**ovalent link, Interfacing residues), ASA (Accessible Surface Area, Å²), BSA (Buried Surface Area, Å²), Δ^i^G (Solvation energy effect, kcal/mol), |||| (Buried area percentage, one bar per 10%).
(DOCX)

S3 Table Mutation sites of SARS-CoV-2 spike variants constructed in this study. (DOCX)
