# Supplementary figures and images for: Structural basis and mode of action for two broadly neutralizing nanobodies targeting the highly conserved spike stem-helix of sarbecoviruses including SARS-CoV-2 and its variants
Source: PLoS Pathog. 2025 Apr 11;21(4):e1013034. doi: 10.1371/journal.ppat.1013034 (PMC12052392; doi:10.1371/journal.ppat.1013034)

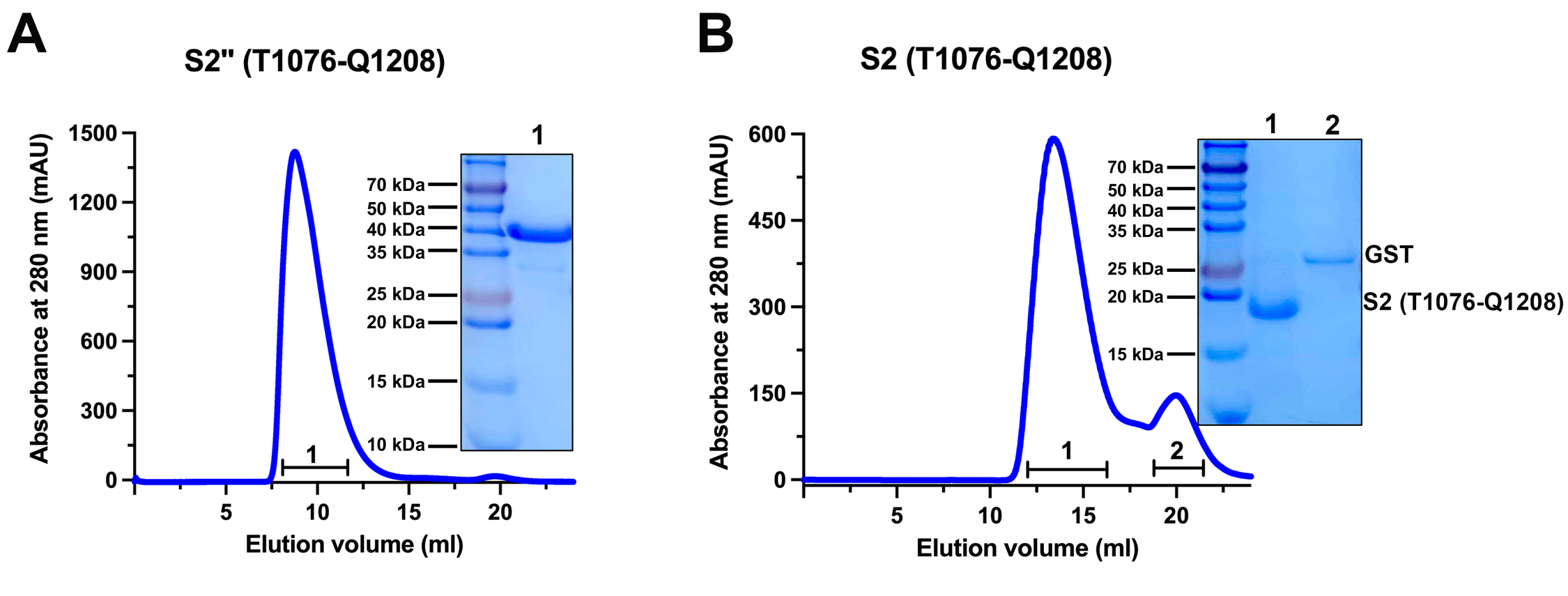

Supplement: S1 Fig — Solution behaviors of SARS-CoV-2 S2’‘ (T1076-Q1208) protein before (A) or after (B) PSP digestion on a Superdex 200 Increase 10/300 GL column. Inset figures show the SDS-PAGE analyses of the pooled samples. S2’‘ (T1076-Q1208) protein is fused with GST at the N terminus and T4 fibritin with His at the C terminus. (TIF) [file ppat.1013034.s001.tif]

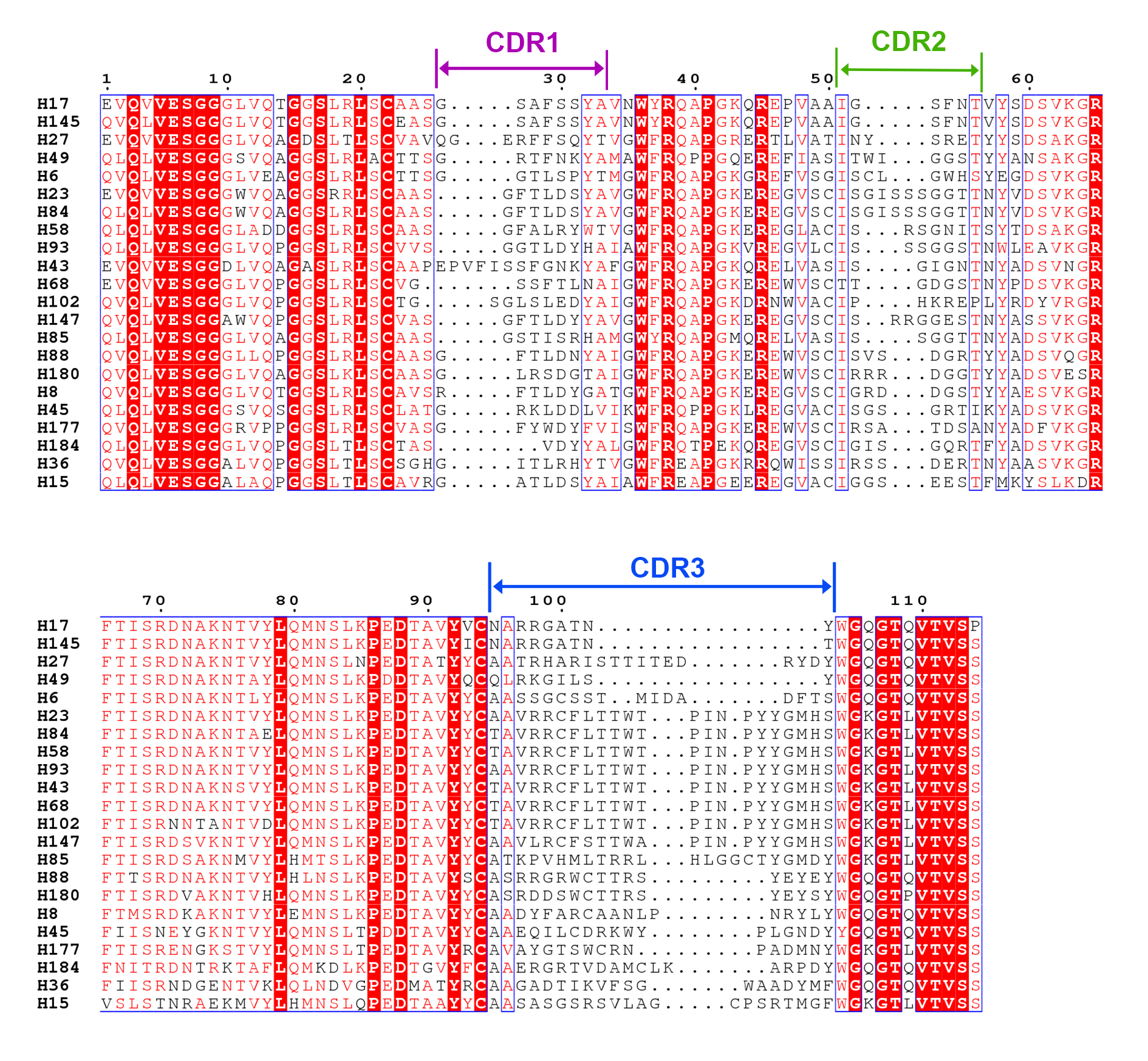

Supplement: S2 Fig — The CDR regions are marked. (TIF) [file ppat.1013034.s002.tif]

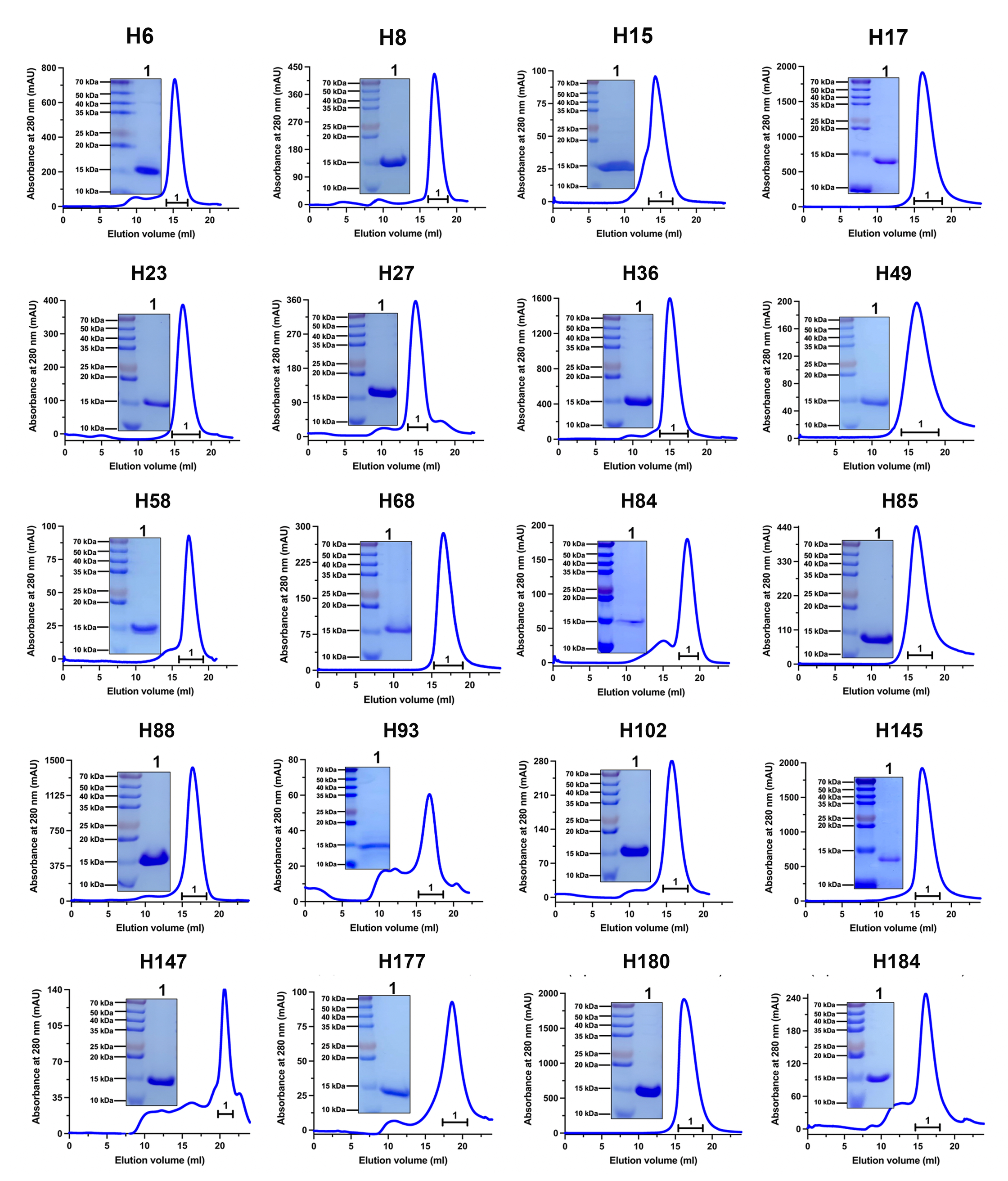

Supplement: S3 Fig — Inset figures show the SDS-PAGE analyses of the pooled samples. (TIF) [file ppat.1013034.s003.tif]

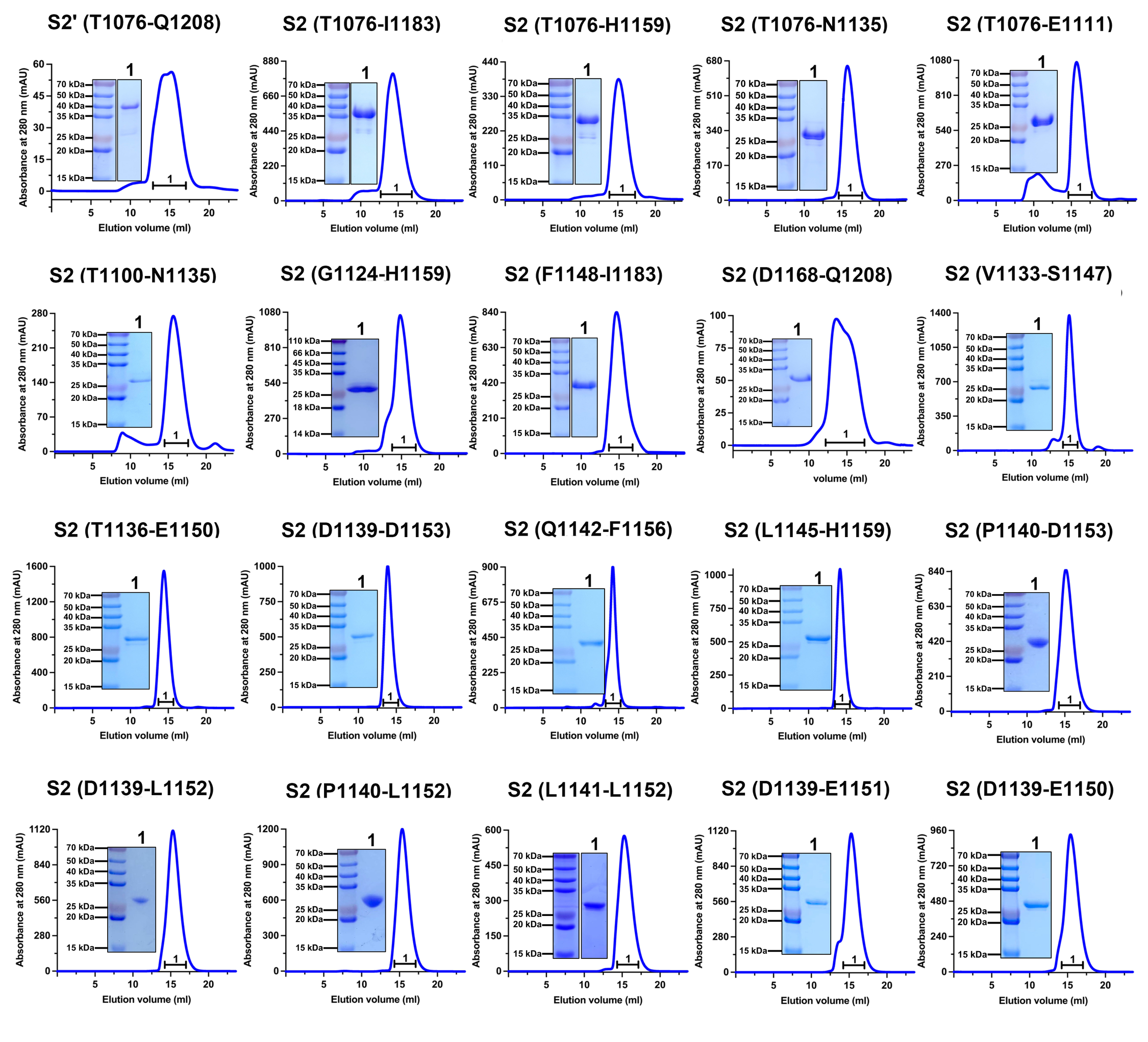

Supplement: S4 Fig — Inset figures show the SDS-PAGE analyses of the pooled samples. These truncated proteins are fused with GST tag at the N terminus. (TIF) [file ppat.1013034.s004.tif]

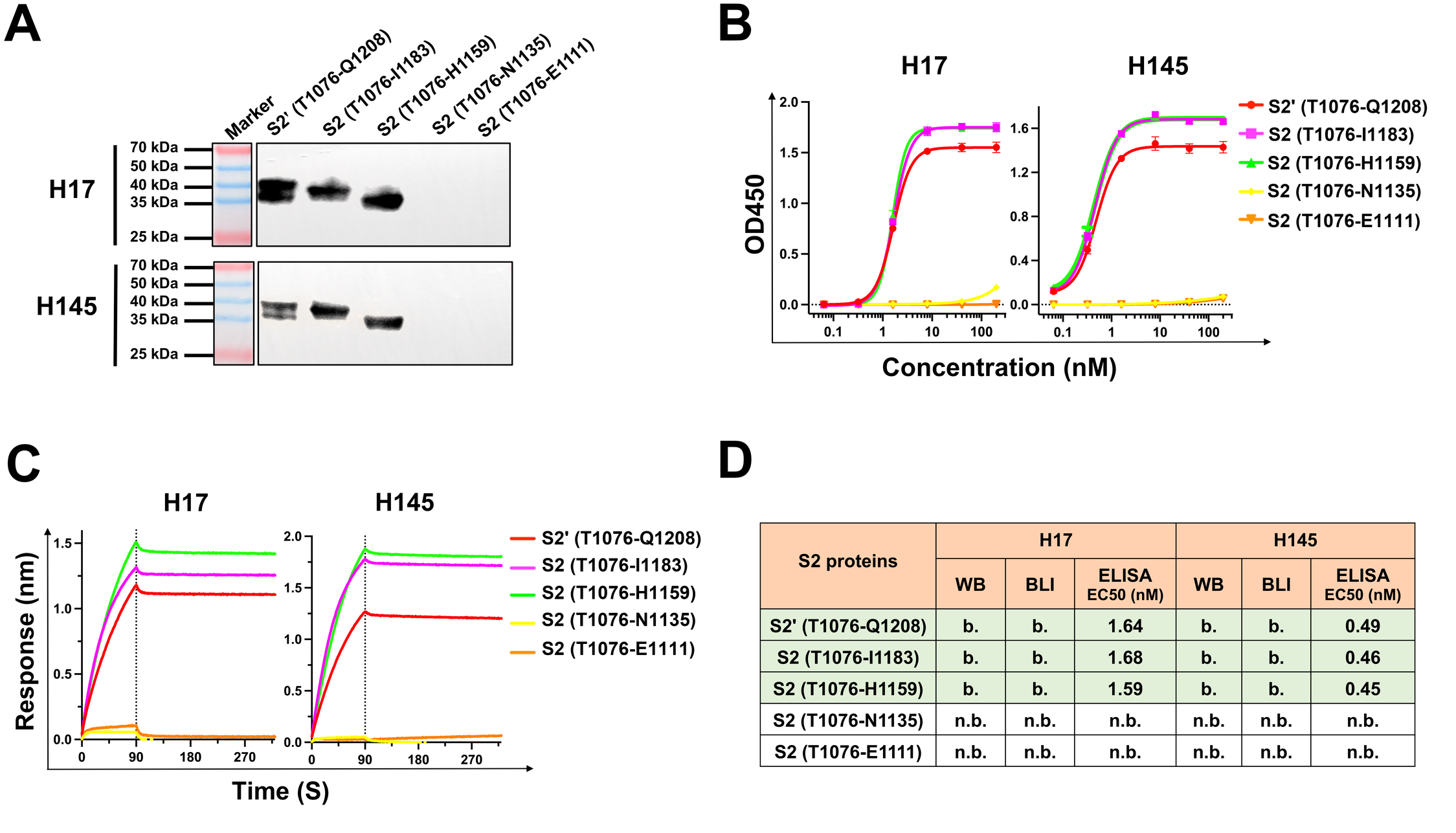

Supplement: S5 Fig — (A) H17 and H145 recognize a linear epitope S2 (T1076-H1159) detected by WB. (B) Multi-concentration ELISA-binding profile of H17 or H145 to the indicated S2 antigen. OD450 emissions are plotted as curves. Data are means ± SD of triplicate samples. (C) Binding ability of the indicated S2 antigen to H17 or H145 analyzed by BLI. Immobilized biotinylated H17 or H145 was saturated in binding with the indicated S2 proteins. Single association and dissociation curves were detected. (D) Summary of the binding features of H17 and H145 to S2-related truncated proteins detected by WB, ELISA, and BLI. b., binding; n.b., no binding. (TIF) [file ppat.1013034.s005.tif]

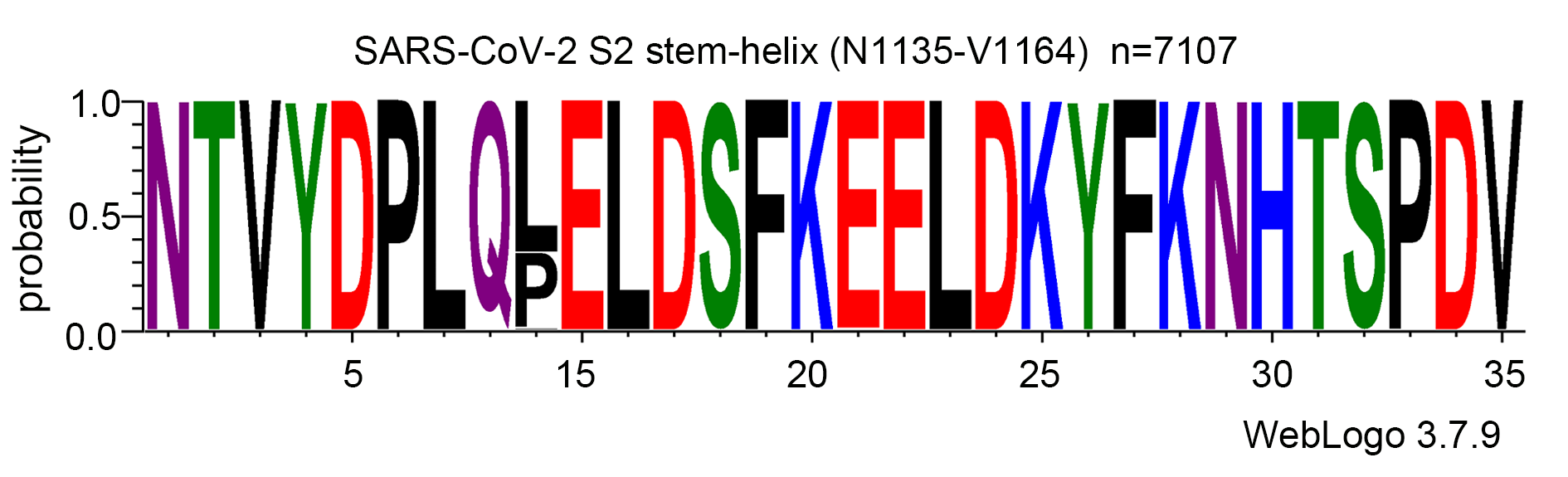

Supplement: S6 Fig — The amino acids of the reported SARS-CoV-2 spike protein was retrieved from the NCBI database and removed erroneous sequences using MEGA7 software, resulting in a final dataset of 7,107 protein sequences. The sequence conservation of the total stem-helix amino acid sequence (N1135-V1164) was conducted using WebLogo (https://weblogo.threeplusone.com). The overall height of the stack indicates the sequence conservation at that position, while the height of symbols within the stack indicates the relative frequency of each amino acid at that position. (TIF) [file ppat.1013034.s006.tif]

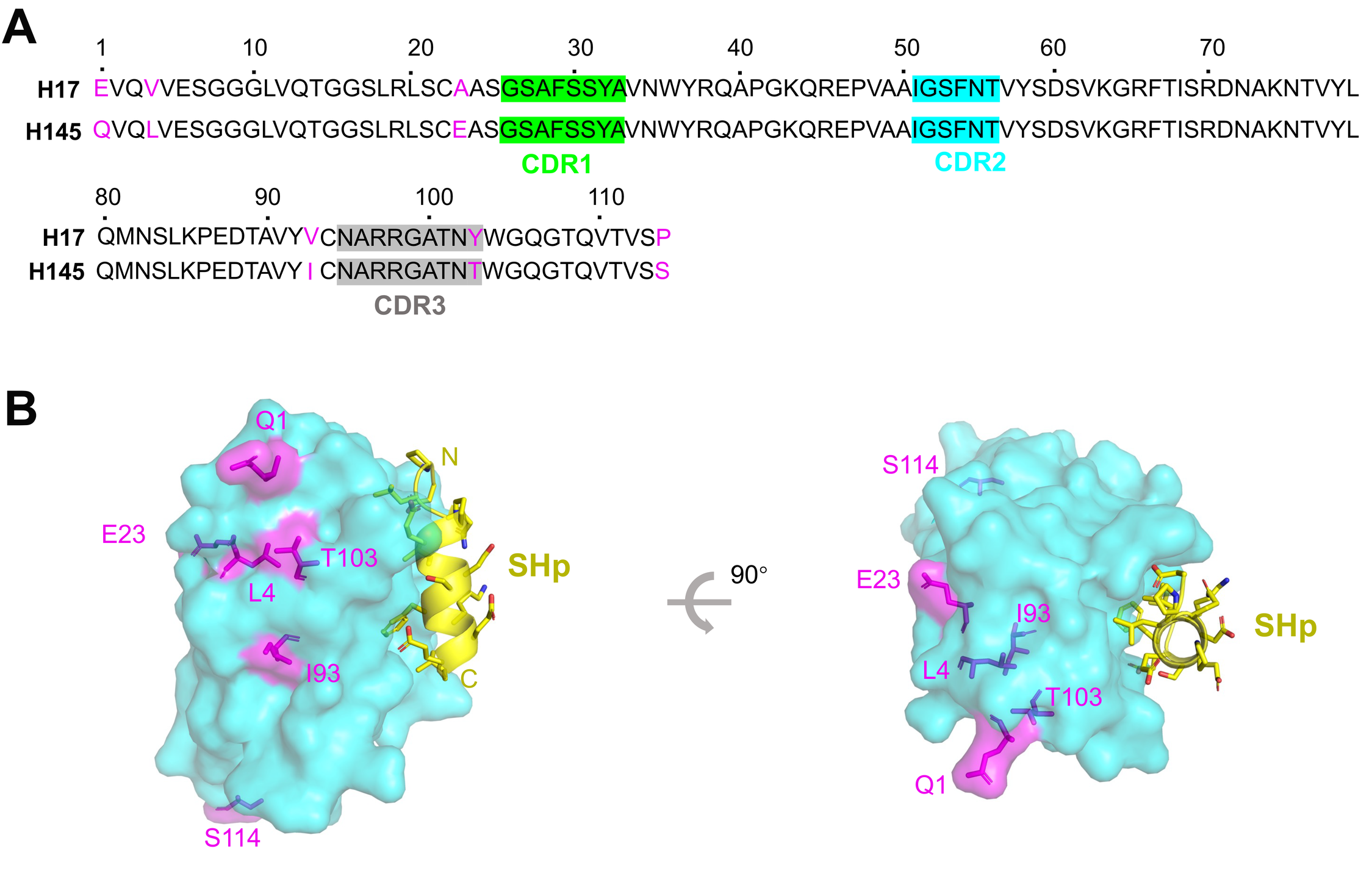

Supplement: S7 Fig — (A) Amino-acid sequence of H17 and H145. Different residues are shown in purple. CDR regions are marked. (B) Six residues in H145 that are different from H17 are shown in the crystal structure of the H145/SH-peptide complex in purple. H145 is shown as surface in aquamarine, and SH-peptide is shown as cartoon in yellow. Side view (left) and top view (right) of structures are presented and shown. (TIF) [file ppat.1013034.s007.tif]

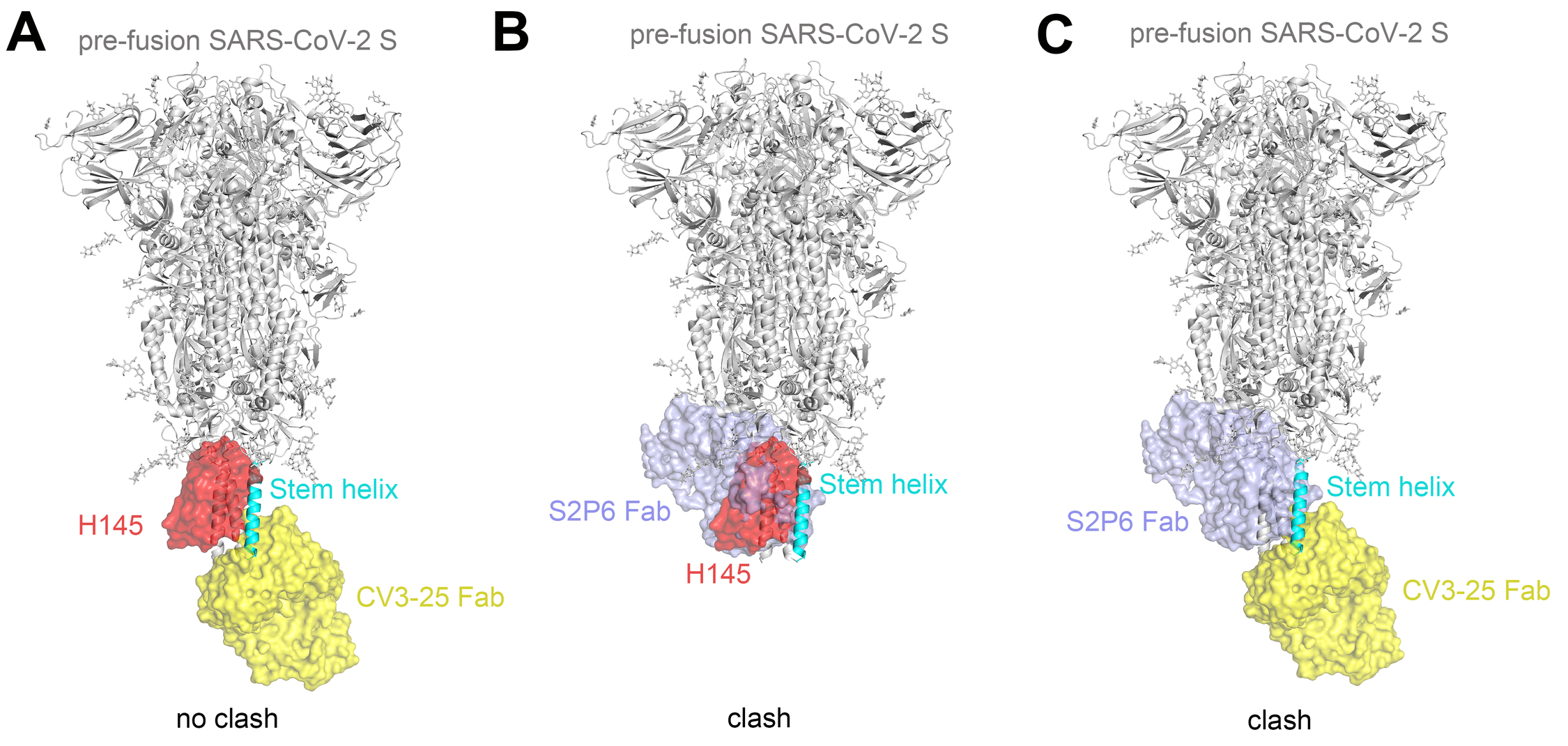

Supplement: S8 Fig — (A-C) Superimposed structures of three antibody fragment/SH-peptide complexes (surface representation) bound to the prefusion SARS-CoV-2 S-trimer (gray cartoon, PDB: 6XR8). (A) H145/SH-peptide complex (PDB: 9LDS reported in this study), colored red. (B) S2P6 Fab/SH-peptide complex (PDB: 7RNJ), colored lightblue. (C) CV3–25 Fab/SH-peptide complex (PDB: 7NAB), colored yellow. In all panels, the stem-helix of one S-trimer monomer is highlighted in cyan (residues D1139-P1162). (TIF) [file ppat.1013034.s008.tif]

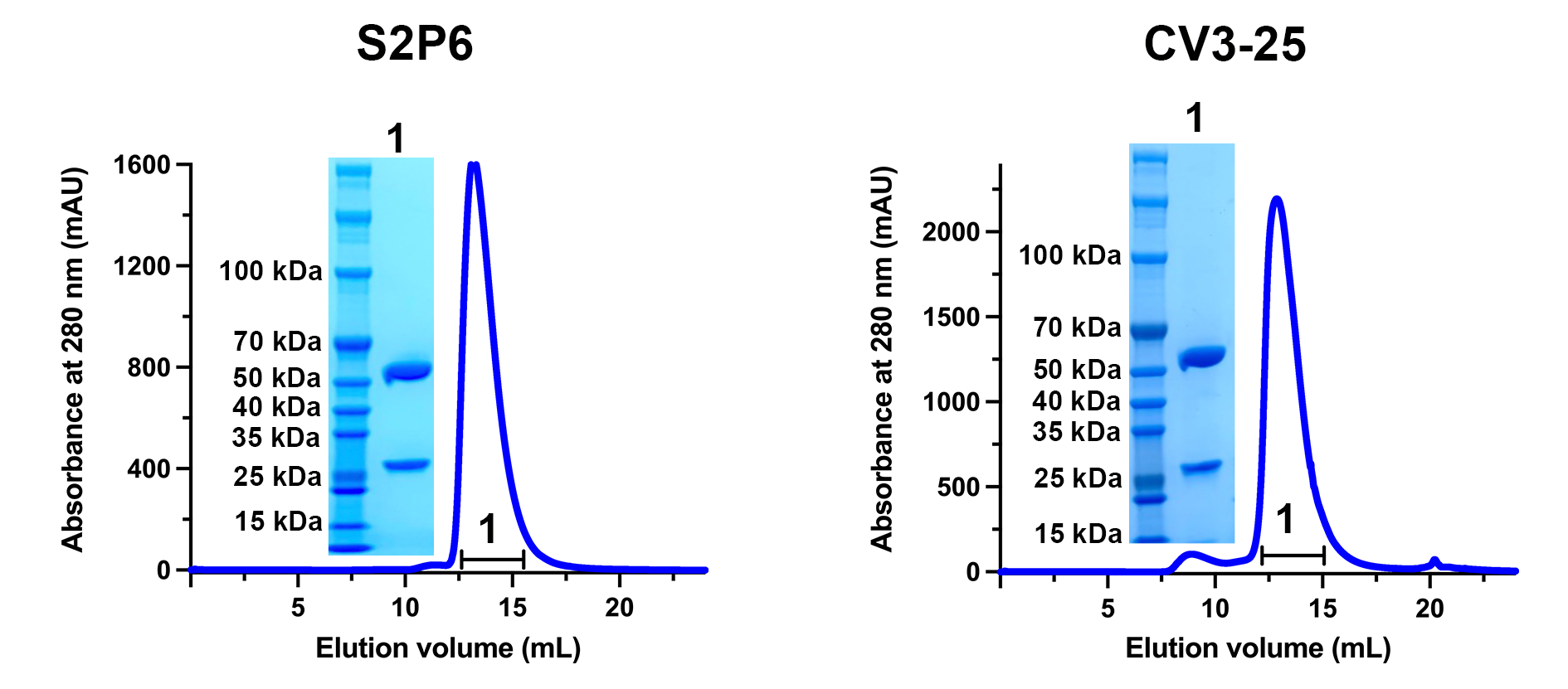

Supplement: S9 Fig — Inset figures show the SDS-PAGE analyses of the pooled samples. (TIF) [file ppat.1013034.s009.tif]

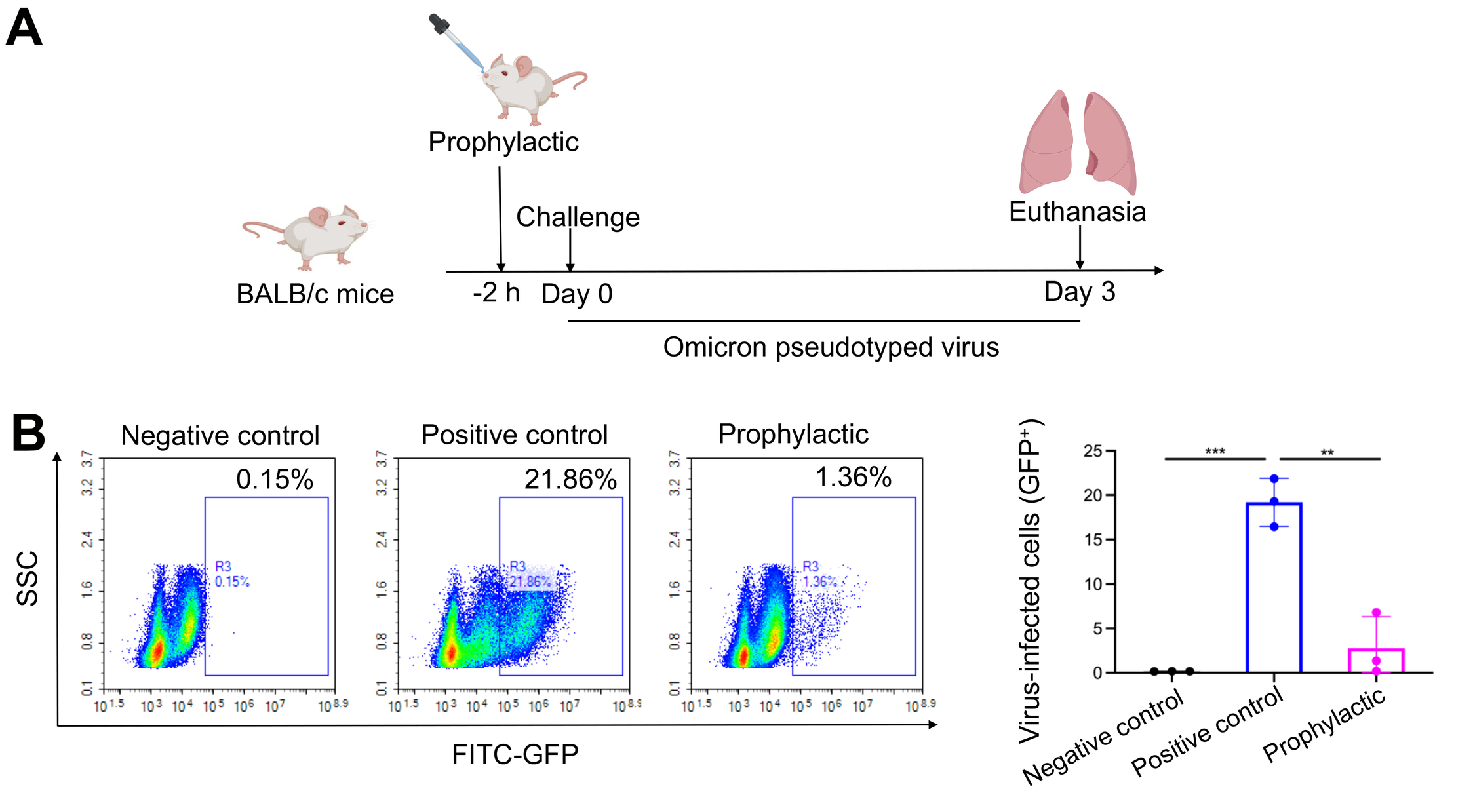

Supplement: S10 Fig — (A) Experimental design for evaluating H145 prophylaxis. Six-week-old female BALB/c mice received intranasal administration of H145 (10 mg/kg) or PBS 2 hours prior to intrathoracic challenge with 4×10^8^ relative light unit (RLU) SARS-CoV-2 Omicoron pseudotyped virus (GFP-Luciferase). Negative controls received PBS without viral challenge. This figure was created using BioRender.com. (B) Virus-infected cells in lung tissues at 3 days post-challenge, quantified by flow cytometry analysis of tissue homogenates. Data are presented as means ± SEM. Statistical significance was determined by unpaired t-test (**P<0.01, ***P<0.001). (TIF) [file ppat.1013034.s010.tif]
