# Supplementary material for: Structural basis and mode of action for two broadly neutralizing nanobodies targeting the highly conserved spike stem-helix of sarbecoviruses including SARS-CoV-2 and its variants
Source: PLoS Pathog. 2025 Apr 11;21(4):e1013034. doi: 10.1371/journal.ppat.1013034 (PMC12052392; doi:10.1371/journal.ppat.1013034)
Supplement: S1 Table — In each case, a single crystal was used to collect the data. Values in parentheses are for the highest-resolution shell. (DOCX) [file ppat.1013034.s011.docx]

**S1 Table. Data collection and structure refinement statistics**

|  | H145/SH-peptide complex structure |
| --- | --- |
| **Data collection** |  |
| Space group | P21 |
| Cell dimensions |  |
| *a*, *b*, *c* (Å) | 43.07, 63.54, 56.06 |
| α, β, γ (°) | 90.00, 112.75, 90.00 |
| Wavelength (Å) | 0.97915 |
| Resolution (Å) | 50.00-1.60 (1.66-1.60) |
| *R*_merge_ | 0.106 (0.596) |
| *I* / sig*I* | 22.519 (2.941) |
| Completeness (%) | 100.0 (99.9) |
| Redundancy | 6.5 (6.0) |
|  |  |
| **Refinement** |  |
| Resolution (Å) | 40.104-1.603 |
| No. reflections | 36580 |
| *R*_work_ / *R*_free_ | 0.1911/0.2020 |
| No. atoms | 2174 |
| Protein | 1963 |
| Water | 211 |
| *B*-factors |  |
| Protein | 16.735 |
| Water | 29.732 |
| R.m.s. deviations |  |
| Bond lengths (Å) | 0.0057 |
| Bond angles (°) | 0.81 |
| Ramachandran plot (%)  Favored region  Allowed region  Outlier region | 98.40  1.60  0.00 |

In each case, a single crystal was used to collect the data. Values in parentheses are for the highest-resolution shell.
