# Supplementary material for: Structural basis and mode of action for two broadly neutralizing nanobodies targeting the highly conserved spike stem-helix of sarbecoviruses including SARS-CoV-2 and its variants
Source: PLoS Pathog. 2025 Apr 11;21(4):e1013034. doi: 10.1371/journal.ppat.1013034 (PMC12052392; doi:10.1371/journal.ppat.1013034)
Supplement: S2 Table — Surface area of H145 on the SARS-CoV-2 S2 stem-helix is shown. The buried areas were calculated with Proteins, Interfaces, Structures and Assemblies (PDBePISA). HSDC (Residues making Hydrogen/Disulphide bond, Salt bridge or Covalent link, Interfacing residues), ASA (Accessible Surface Area, Å²), BSA (Buried Surface Area, Å²), ΔiG (Solvation energy effect, kcal/mol), |||| (Buried area percentage, one bar per 10%). (DOCX) [file ppat.1013034.s012.docx]

**S2 Table. The interface residues in the H145/SH-peptide complex.**

| **SH peptide** | **HSDC** | **ASA** | **BSA** | **Δ^i^G** |
| --- | --- | --- | --- | --- |
| ASP1139 | H | 115.23 | 74.86 \|\|\|\|\|\|\| | -0.14 |
| PRO1140 |  | 132.27 | 26.82 \|\|\| | 0.43 |
| LEU1141 |  | 110.90 | 98.56 \|\|\|\|\|\|\|\|\| | 1.58 |
| GLN1142 | H | 68.94 | 14.32 \|\|\| | -0.24 |
| PRO1143 |  | 91.34 | 0.00 | 0.00 |
| GLU1144 | H | 99.85 | 74.81 \|\|\|\|\|\|\|\| | 0.35 |
| LEU1145 |  | 84.46 | 83.62 \|\|\|\|\|\|\|\|\|\| | 1.32 |
| ASP1146 |  | 60.32 | 0.00 | 0.00 |
| SER1147 | H | 58.49 | 19.88 \|\|\|\| | -0.13 |
| PHE1148 |  | 119.63 | 119.63 \|\|\|\|\|\|\|\|\|\| | 1.91 |
| LYS1149 |  | 115.06 | 21.08 \|\| | 0.34 |
| GLU1150 |  | 124.08 | 0.00 | 0.00 |
| GLU1151 | HS | 130.34 | 56.82 \|\|\|\|\| | -0.59 |
| LEU1152 |  | 138.37 | 70.92 \|\|\|\|\|\| | 1.13 |
| ASP1153 |  | 151.53 | 0.00 | 0.00 |

Surface area of H145 on the SARS-CoV-2 S2 stem-helix is shown. The buried areas were calculated with Proteins, Interfaces, Structures and Assemblies (PDBePISA). HSDC (Residues making **H**ydrogen/**D**isulphide bond, **S**alt bridge or **C**ovalent link, Interfacing residues), ASA (Accessible Surface Area, Å²), BSA (Buried Surface Area, Å²), Δ^i^G (Solvation energy effect, kcal/mol), |||| (Buried area percentage, one bar per 10%).
