# Supplementary material for: Structural basis and mode of action for two broadly neutralizing nanobodies targeting the highly conserved spike stem-helix of sarbecoviruses including SARS-CoV-2 and its variants
Source: PLoS Pathog. 2025 Apr 11;21(4):e1013034. doi: 10.1371/journal.ppat.1013034 (PMC12052392; doi:10.1371/journal.ppat.1013034)
Supplement: S3 Table — (DOCX) [file ppat.1013034.s013.docx]

**S3 Table.** **Mutation sites of SARS-CoV-2 spike variants constructed in this study.**

| **Variants** | | **Mutation sites** | |
| --- | --- | --- | --- |
| BA.1 | Omicron | A67V, Δ69-70, T95I, G142D/Δ143-145, Δ211/L212I, ins214EPE, G339D, S371L, S373P, S375F, K417N, N440K, G446S, S477N, T478K, E484A, Q493R, G496S, Q498R, N501Y, Y505H, T547K, D614G, H655Y, N679K, P681H, N764K, D796Y, N856K, Q954H, N969K, L981F |  |
| BA.2 | Omicron | T19I, L24S, (del 25-27) 25PPAins, G142D, V213G, G339D, S371F, S373P, S375F, T376A, D405N, R408S, K417N, N440K, S477N, T478K, E484A, Q493R, Q498R, N501Y, Y505H, D614G, H655Y, N679K, P681H, N764K, D796Y, Q954H, N969K |  |
| BA.4&5 | Omicron | T19I, L24S, del25-del27, del69, del70, G142D, V213G, G339D, S371F, S373P, S375F, T376A, D405N, R408S, K417N, N440K, L452R, S477N, T478K, E484A, F486V, Q498R, N501Y, Y505H, D614G, H655Y, N679K, P681H, N764K, D796Y, Q954H, N969K |  |
| BA.2.12.1 | Omicron | T19I, L24S, (del25-27) 25PPAins, G142D, V213G, G339D, S371F, S373P, S375F, T376A, D405N, R408S, K417N, N440K, L452Q, S477N, T478K, E484A, Q493R, Q498R, N501Y, Y505H, D614G, H655Y, N679K, P681H, S704L, N764K, D796Y, Q954H, N969K |  |
| BF.7 | Omicron | T19I, L24S, del25-del27, del69, del70, G142D, V213G, G339D, R346T, S371F, S373P, S375F, T376A, D405N, R408S, K417N, N440K, L452R, S477N, T478K, E484A, F486V, Q498R, N501Y, Y505H, D614G, H655Y, N679K, P681H, N764K, D796Y, Q954H, N969K |  |
| BA.2.75 | Omicron | T19I, LPP24-26del, A27S, K147E, W152R, F157L, I210V, V213G, G257S, G339H, S371F, S373P, S375F, T376A, D405N, R408S, K417N, N440K, G446S, N460K, S477N, T478K, E484A, Q498R, N501Y, Y505H, D614G, H655Y, N679K, P681H, N764K, D796Y, Q954H, N969K |  |
| XBB.1.5 | Omicron | T19I, LPP24-26del, A27S, V83A, G142D, Y144del, H146Q, Q183E, V213E, G252V, G339H, R346T, L368I, S371F, S373P, S375F, T376A, D405N, R408S, K417N, N440K, V445P, G446S, N460K, S477N, T478K, E484A, F486P, F490S, Q498R, N501Y, Y505H, D614G, H655Y, N679K, P681H, N764K, D796Y, Q954H, N969K |  |
| XBB.1.16 | Omicron | T19I, V83A, G142D, H146Q, E180V, Q183E, V213E, G339H, R346T, L368I, S371F, S373P, S375F, T376A, D405N, K417N, N440K, V445P, G446S, N460K, S477N, T478R, E484A, F486P, F490S, Q498R, N501Y, Y505H, D614G, H655Y, N679K, P681H, N764K, D796Y, Q954H, N969K |  |
| JN.1 | Omicron | T19I, R21T, L24S, Del25-27, S50L, Del69-70, V127F, G142D, Del144, F157S, R158G, N211I, Del212, V213G, L216F, H245N, A264D, I332V, G339H, K356T, S371F, S373P, S375F, T376A, R403K, D405N, R408S, K417N, N440K, V445H, G446S, N450D, L452W, L455S, N460K, S477N, T478K, N481K, Del483, E484K, F486P, Q498R, N501Y, Y505H, E554K, A570V, D614G, P621S, H655Y, N679K, P681R, N764K, D796Y, S939F, Q954H, N969K, P1143L |  |
| KP.3 | Omicron | T19I, R21T, L24S, DEL25-27, S50L, DEL69-70, V127F, G142D, DEL144, F157S, R158G, N211I, DEL212, V213G, L216F, H245N, A264D, I332V, G339H, K356T, S371F, S373P, S375F, T376A, R403K, D405N, R408S, K417N, N440K, V445H, G446S, N450D, L452W, L455S, F456L, N460K, S477N, T478K, N481K, DEL483, E484K, F486P, Q493E, Q498R, N501Y, Y505H, E554K, A570V, D614G, P621S, H655Y, N679K, P681R, N764K, D796Y, S939F, Q954H, N969K, V1104L, P1143L |  |
| B.1.351 | Beta | L18F, D80A, D215G, L242-244del, R246I, K417N, E484K , N501Y, D614G, A701V |  |
| P.1 | Gamma | L18F, T20N, P26S, D138Y, R190S, K417T, E484K, N501Y, D614G, H655Y, T1027I, V1176F |  |
| B.1.617.1 | Kappa | T95I, G142D, E154K, L452R, E484Q, D614G, P681R, Q1071H |  |
| B.1.617.2 | Delta | T19R, G142D, del156-157, R158G, L452R, T478K, D614G, P681R, D950N |  |
| B.1.621 | Mu | T95I, Y144S, Y145N, R346K, E484K, N501Y, D614G, P681H, D950N |  |
